# Verapamil mitigates chloride and calcium bi-channelopathy in a myotonic dystrophy mouse model

**DOI:** 10.1172/JCI173576

**Published:** 2024-01-02

**Authors:** Lily A. Cisco, Matthew T. Sipple, Katherine M. Edwards, Charles A. Thornton, John D. Lueck

**Affiliations:** 1 Department of Pharmacology and Physiology,; 2 Department of Neurology,; 3 Center for RNA Biology, University of Rochester School of Medicine and Dentistry, Rochester, New York, USA.

**Keywords:** Muscle Biology, Therapeutics, Calcium channels, Chloride channels, Muscle

## Abstract

Myotonic dystrophy type 1 (DM1) involves misregulated alternative splicing for specific genes. We used exon or nucleotide deletion to mimic altered splicing of genes central to muscle excitation-contraction coupling in mice. Mice with forced skipping of exon 29 in the Ca_V_1.1 calcium channel combined with loss of ClC-1 chloride channel function displayed markedly reduced lifespan, whereas other combinations of splicing mimics did not affect survival. The Ca^2+^/Cl^–^ bi-channelopathy mice exhibited myotonia, weakness, and impairment of mobility and respiration. Chronic administration of the calcium channel blocker verapamil rescued survival and improved force generation, myotonia, and respiratory function. These results suggest that Ca^2+^/Cl^–^ bi-channelopathy contributes to muscle impairment in DM1 and is potentially mitigated by common clinically available calcium channel blockers.

## Abstract 2

Repurposing of a calcium channel blocker extends life and mitigates muscle and respiratory dysfunction in a myotonic dystrophy type 1 Ca2+/Cl- bi-channelopathy mouse model.

## Introduction

Myotonic dystrophy type 1 (DM1) is an autosomal dominant disorder characterized by myotonia, progressive weakness, heart block, gastrointestinal dysmotility, predisposition to malignancy, and other symptoms (1). Survival is limited by weakness of the respiratory muscles or cardiac arrhythmia. The genetic basis is an expansion of CTG repeats in the 3′-UTR of the *DM1* protein kinase (*DMPK*) gene. Although DM1 can begin in infancy, more often the symptoms are delayed until the second to fifth decades. The onset and progression of DM1 is believed to reflect an age-dependent increase in the size of expanded repeats in tissue, reaching lengths of several thousand repeats in skeletal and cardiac muscle.

The core mechanism for DM1 is RNA toxicity, whereby *DMPK* transcripts with expanded CUG repeats form nuclear condensates that sequester splicing factors in the muscleblind-like (MBNL) family. The resulting loss of MBNL function causes misregulation of alternative splicing and other changes in RNA processing. This in turn produces a complex array of clinical findings in which specific endophenotypes, such as myotonia, heart block, or insulin resistance, are linked to splicing defects of particular genes, such as *CLCN1* (chloride channel) (2–4), *SCN5A* (sodium channel) (5, 6), or *INSR* (insulin receptor) (7).

Efforts to deconstruct the DM1 phenotype have taken a reductionist approach, in which individual splicing defects are studied in isolation (8, 9). However, the main source of disability — progressive muscle weakness — remains largely unexplained. It is unclear whether motor disability results from predominant effects of an individual splicing defect or combinatorial mis-splicing of several or many transcripts. Although splicing misregulation of many alternative exons correlates with muscle weakness, it has been difficult to establish causal relationships because the splicing changes are highly intercorrelated (10). Accordingly, we used exon or nucleotide deletion to mimic splicing defects affecting 4 genes involved in sequential steps of excitation-contraction coupling (ECC), including *Clcn1* (encoding ClC-1 chloride channel), *Cacna1s* (Ca_V_1.1 calcium channel, dihydropyridine receptor), *Ryr1* (ryanodine receptor, Ca^2+^ release channel), and *Atp2a1* (sarcoplasmic reticulum Ca^2+^-ATPase, SERCA1, Ca^2+^ reuptake pump). Then, we used breeding to examine combinatorial effects. We found that ClC-1/Ca_V_1.1 bi-channelopathy, but not other combinations, was highly deleterious, causing weakness, respiratory impairment, and a marked reduction of lifespan. The survival of bi-channelopathy mice was dramatically improved by chronic oral feeding of the L-type calcium channel blocker verapamil. These results support a contributory role for excess calcium entry in DM1 pathogenesis and suggest that Ca^2+^ channel modulation is a potential therapeutic strategy.

## Results

### Generation of mouse models with forced mis-splicing.

**Figure 1:**
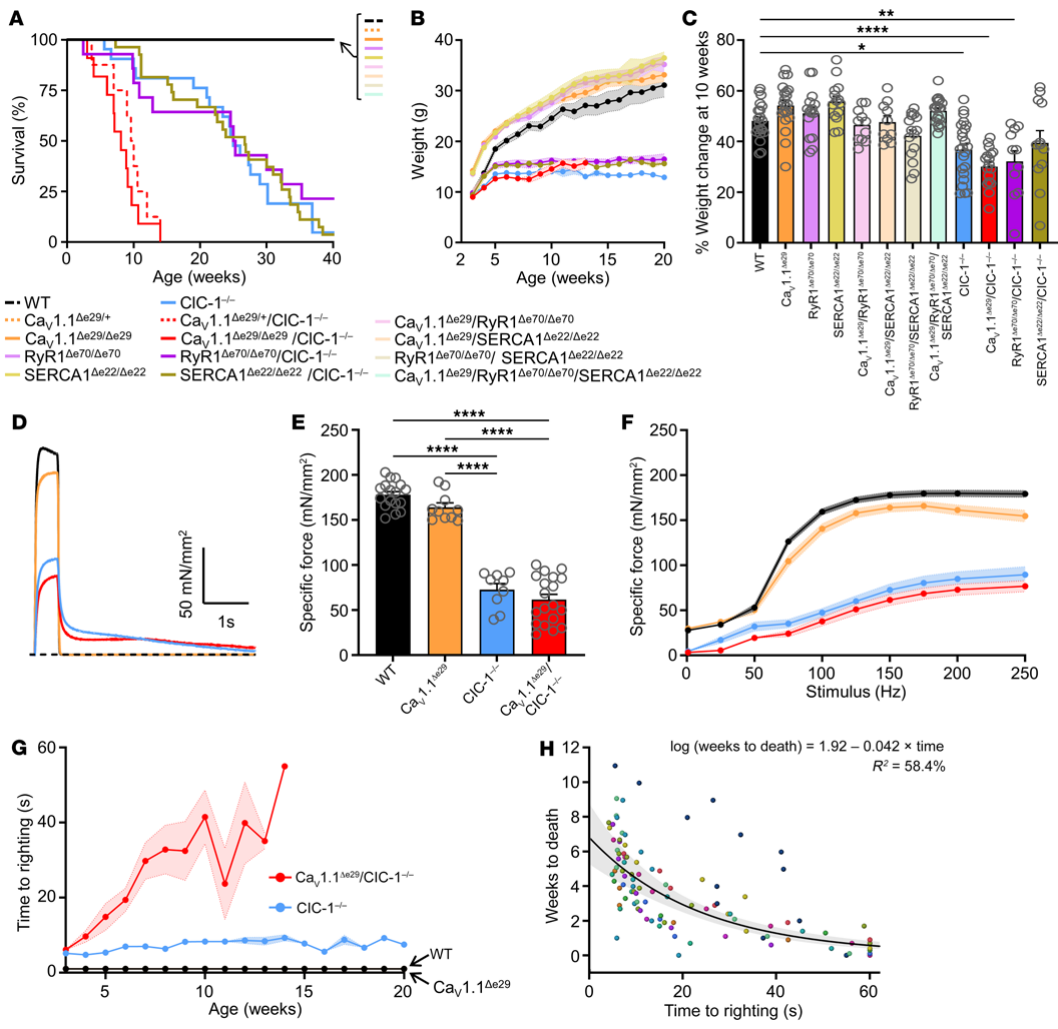
Ca_V_1.1^Δe29^ and ClC-1^–/–^ alleles exhibit synthetic lethality and result in significantly reduced body weight and severe muscle weakness in mice. (**A**) Kaplan-Meier survival analysis of WT (*n* = 10; female = 5, male = 5), Ca_V_1.1^Δe29/+^ (*n* = 13; female = 7, male = 6), Ca_V_1.1^Δe29^ (*n* = 15; female = 7, male = 8), RyR1^Δe70/Δe70^ (*n* = 21; female = 10, male = 11), SERCA1^Δe22/Δe22^ (*n* = 19; female = 10, male = 9), Ca_V_1.1^Δe29^ RyR1^Δe70/Δe70^ (*n* = 11; female = 5, male = 6), Ca_V_1.1^Δe29^ SERCA1^Δe22/Δe22^ (*n* = 10; female = 6, male = 4), RyR1^Δe70/Δe70^ SERCA1^Δe22/Δe22^ (*n* = 15; female = 4, male = 11), Ca_V_1.1^Δe29^ RyR1^Δe70/Δe70^ SERCA1^Δe22/Δe22^ (*n* = 19; female = 6, male = 13), ClC-1^–/–^ (*n* = 27; female = 12, male = 15), RyR1^Δe70/Δe70^ ClC-1^–/–^ (*n* = 14; female = 7, male = 7), SERCA1^Δe22/Δe22^ ClC-1^–/–^ (*n* = 27; female = 14, male = 13), Ca_V_1.1^Δe29/+^ ClC-1^–/–^ (*n* = 8; female = 3, male = 5), and Ca_V_1.1^Δe29/Δe29^ ClC-1^–/–^ (*n* = 11; female = 6, male = 5) mice. (**B**) Weekly body weight change and (**C**) percentage of body weight change from weaning at 10 weeks of age for WT (*n* = 20; female = 10, male = 10), Ca_V_1.1^Δe29^ (*n* = 21; female = 12, male = 9), RyR1^Δe70/Δe70^ (*n* = 15; female = 5, male = 10), SERCA1^Δe22/Δe22^ (*n* = 14; female = 7, male = 7), Ca_V_1.1^Δe29^ RyR1^Δe70/Δe70^ (*n* = 11; female = 5, male = 6), Ca_V_1.1^Δe29^ SERCA1^Δe22/Δe22^ (*n* = 10; female = 6, male = 4), RyR1^Δe70/Δe70^ SERCA1^Δe22/Δe22^ (*n* = 15; female = 4, male = 11), Ca_V_1.1^Δe29^ RyR1^Δe70/Δe70^ SERCA1^Δe22/Δe22^ (*n* = 19; female = 6, male = 13), ClC-1^–/–^ (*n* = 23; female = 13, male = 10), Ca_V_1.1^Δe29^ ClC-1^–/–^ (*n* = 17; female = 8, male = 9), RyR1^Δe70/Δe70^ ClC-1^–/–^ (*n* = 11; female = 5, male = 6), and SERCA1^Δe22/Δe22^ ClC-1^–/–^ (*n* = 12; female = 8, male = 4) mice. (**D**) Representative traces and (**E**) average peak specific force elicited by 150 Hz (500 ms) tetanic stimulation of isolated EDLs from mice at 10 weeks. (**F**) Average frequency dependence of specific force generation elicited from isolated EDLs at 10 weeks. (**E** and **F**) WT (*n* = 17; female = 8, male = 9), Ca_V_1.1^Δe29^ (*n* = 10; female = 5, male = 5), ClC-1^–/–^ (*n* = 9; female = 4, male = 5), Ca_V_1.1^Δe29^ ClC-1^–/–^ (*n* = 19; female = 8, male = 11) mice were used. (**G**) Weekly TRR analysis of Ca_V_1.1^Δe29^ ClC-1^–/–^ (red), ClC-1^–/–^ (blue), Ca_V_1.1^Δe29^ (orange), WT (black) mice. (**H**) Correlation of the TRR to death for Ca_V_1.1^Δe29^ ClC-1^–/–^ mice. Dots represent individual mice, and duplications indicate TRR trials. In **B**–**H**, results in red represent heterozygous and homozygous Ca_V_1.1^Δe29^ mice that are ClC-1^–/–^, and results in orange indicate heterozygous and homozygous Ca_V_1.1^Δe29^ alleles. Symbols and open circles indicate individual mice; bars and closed circles indicate the mean ± SEM. *P < 0.05, **P < 0.01, and ****P < 0.0001, by log-rank analysis (**A**); 2-way ANOVA (**B**, **F**, and **G**) and 1-way ANOVA (**C** and **E**) with Tukey’s post hoc analysis; and linear regression analysis (**H**).

ECC stands out among pathways affected by DM1 mis-splicing because 3 sequential components are strongly affected by exon skipping (*CACNA1S* exon 29, *RYR1* exon 70, and *ATP2a1* exon 22), and a fourth shows abnormal exon inclusion (*CLCN1* exon 7a). To reproduce each effect, we generated congenic mice that have genomic deletion of the DM1-skipped exons RyR1^Δe70^, SERCA1^Δe22^, or Ca_V_1.1^Δe29^ and then verified production of the expected splice products by cDNA sequencing. To reproduce effects of *CLCN1* mis-splicing, we used a single-nucleotide deletion (*adr*^mto2j^ mice) that, like exon 7a inclusion, causes frameshift, protein truncation and loss of channel function (ClC-1^–/–^) (11). We found that all splicing mimics studied in isolation, whether in the heterozygous or homozygous state, exhibited normal weight gain and survival, except homozygous ClC-1^–/–^ mice. These mice showed obvious action myotonia (stiffness of limb muscles when stimulated to move after a sedentary interval), reduction of weight gain, and an average survival of 25 weeks (Figure 1A).

Next, we tested for combinatorial effects. Through breeding, we obtained mice doubly homozygous for each possible combination of 2 splicing mimics (*n* = 7 combinations) and for the triple combination of RyR1^Δe70^, SERCA1^Δe22^, and Ca_V_1.1^Δe29^. One combination emerged as highly deleterious. The mean lifespan of Ca_V_1.1^Δe29/Δe29^ ClC-1^–/–^ doubly homozygous mice was 8.1 weeks (Figure 1A). Even with heterozygosity for Ca_V_1.1^Δe29^, the mean lifespan of mice on the ClC-1^–/–^ background was 9.8 weeks, with none of these mice surviving past 14 weeks. As other combinations did not show reduced survival or failure to gain weight that differed from ClC-1^–/–^ mice (Figure 1, B and C), our subsequent experiments, including those outlined in Figure 1, D–H, focused on the Ca^2+^/Cl^–^ bi-channelopathy mice.

**Figure 2:**
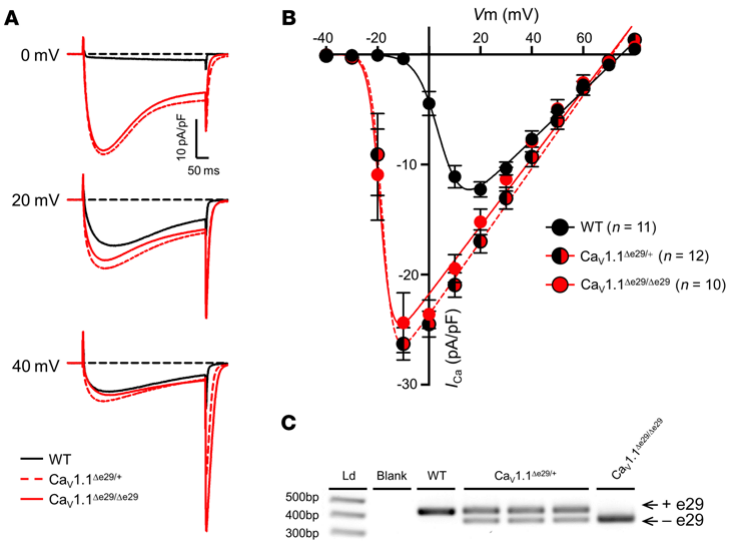
Heterozygous and homozygous Ca_V_1.1^Δe29^ mice exhibit similar Ca_V_1.1 voltage dependence and peak current densities in FDB muscle. (**A**) Representative current density traces from whole-cell patch clamp recordings of FDB fibers isolated from 4-week-old WT (black), Ca_V_1.1^Δe29/+^ (red, dashed), and Ca_V_1.1^Δe29/Δe29^ (red, solid) mice at 0 mV (top), +20 mV (middle), and +40 mV (bottom). (**B**) Plot of average current-voltage relationship of Ca_V_1.1 activity measured in WT (black), Ca_V_1.1^Δe29/+^ (red and black circles, red dashed), and Ca_V_1.1^Δe29/Δe29^ (red circle, solid red line) FDB fibers isolated from 4-week-old mice. (**C**) RT-PCR products of Ca_V_1.1 RNA isolated from tibialis anterior from 10-week-old mice. PCR amplifications are from exons 27–31 of *Cacna1s* cDNA. Ld, ladder. Statistical significance was determined by 1-way ANOVA with Tukey’s post hoc analysis (**B**).

These results indicated that exon 29 skipping for Ca_V_1.1 is highly deleterious in the context of ClC-1 loss and suggested that Ca_V_1.1^Δe29^ channels exert a dominant gain of function. To test the latter possibility, we used whole-cell patch-clamp recording to isolate macroscopic Ca_V_1.1 currents in flexor digitorum brevis (FDB) muscle fibers. As compared with WT littermates, Ca_V_1.1 channel activity in homozygous Ca_V_1.1^Δe29/Δe29^ mouse fibers exhibited a significant leftward shift in activation (~25 mV) and an augmented peak current density (Figure 2, A and B, and Supplemental Table 1; supplemental material available online with this article; https://doi.org/10.1172/JCI173576DS1), comparable to previous findings in homozygous Ca_V_1.1^Δe29/Δe29^ mice and in heterologous expression studies (9, 12). Surprisingly, we found that abnormalities in Ca_V_1.1 channel activity in heterozygous Ca_V_1.1^Δe29/+^ fibers were indistinguishable from channel activity in homozygotes, confirming a dominant gain of function by Ca_V_1.1^Δe29^ channels. Using reverse transcription PCR (RT-PCR) analysis, we found that dominant behavior of Ca_V_1.1^Δe29^ channels in Ca_V_1.1^Δe29/+^ heterozygous mice did not result from unequal allelic accumulation of Ca_V_1.1 mRNA (Figure 2C). Taken together, these results suggest that increased muscle activity (myotonic runs due to ClC-1 loss) plus increased calcium entry during muscle activity (via Ca_V_1.1^Δe29^ channels) caused premature death in bi-channelopathy mice.

Next, we examined the effects of single versus dual channelopathy on ex vivo force generation in hind limb muscle. Since Ca_V_1.1 activity was similar in Ca_V_1.1^Δe29/+^ and Ca_V_1.1^Δe29/Δe29^ mice, these genotypes were grouped together (“Ca_V_1.1^Δe29^”) for these and subsequent analyses and are plotted in the figures in red unless otherwise noted. Single-twitch and tetanic stimulation of extensor digitorum longus (EDL) muscle showed a similar reduction of maximum force in single-channelopathy (ClC-1^–/–^, Figure 1, D–F, blue) and bi-channelopathy (Ca_V_1.1^Δe29^ ClC-1^–/–^, Figure 1, D–F, red) muscle as compared with WT mouse muscle (Figure 1, D–F, black) and Ca_V_1.1^Δe29^ (Figure 1, D–F, orange) controls. Representative traces of tetanic force (150 Hz, Figure 1D) also demonstrated myotonia, as evidenced by delayed muscle relaxation in ClC-1^–/–^ (blue trace) and Ca_V_1.1^Δe29^ ClC-1^–/–^ (red trace) muscle, which was absent in WT (black trace) and Ca_V_1.1^Δe29^ (orange trace) controls.

These results under conditions of ex vivo tetanic stimulation suggested fixed weakness in ClC-1 null muscle, as observed with age in some patients with recessive myotonia congenita due to biallelic mutations of ClC-1 (13), but showed no difference in contractility to account for the premature mortality in bi- versus single-channelopathy mice. Muscle histochemistry and immunofluorescence at 10 weeks showed the expected adaptive response to myotonia in ClC-1^–/–^ muscles, such as fiber type switching and increased oxidative activity, but also failed to distinguish single-channelopathy mice from bi-channelopathy mice. Furthermore, the bi-channelopathy mice did not show a flagrant myopathy or histologic features characteristic of human DM1, such as central nuclei or circumferential myofibrils (ring fibers) (Supplemental Figures 1–5). However, the mobility of bi-channelopathy mice was strikingly affected. A well-known feature of ClC-1–null mice, also known as arrested development of righting response (*adr*) mice, is the slow recovery of upright posture after being placed supine (14–16), and this righting response was clearly delayed in ClC-1^–/–^ mice compared with WT or Ca_V_1.1^Δe29^ control mice (Figure 1G, blue). However, the time of righting response (TRR) was much greater in bi-channelopathy mice, and this deficit was progressive (Figure 1G, red). Indeed, a rise of the righting response beyond 30 seconds was predictive of death or the survival endpoint in bi-channelopathy mice (Figure 1H).

### Ca_V_1.1^Δe29^ conductance potentiates muscle transient weakness in the context of ClC-1 loss.

**Figure 3:**
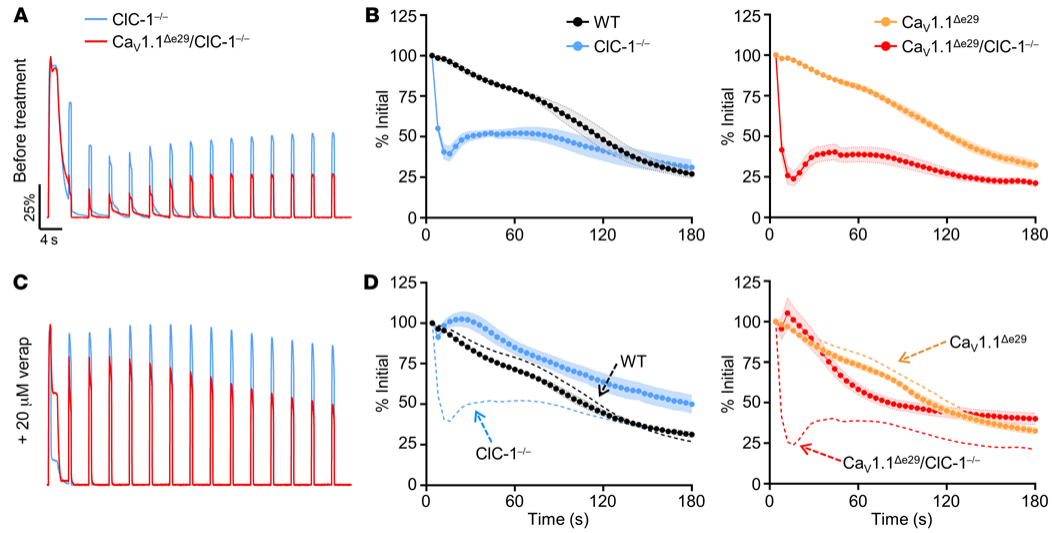
Ca_V_1.1^Δe29^ ClC-1^–/–^ and ClC-1^–/–^ muscle exhibits severe transient weakness that is significantly improved by the addition of verapamil. (**A** and **C**) Normalized representative force traces of the first 15 tetani (100 Hz, 500 ms) separated by 4 seconds, recorded ex vivo from EDL muscles isolated from 6-week-old ClC-1^–/–^ (blue) and Ca_V_1.1^Δe29^ ClC-1^–/–^ (red) mice in the (**A**) absence and (**C**) presence of 20 μM verapamil added to the bath. (**B** and **D**) Average peak tetanic EDL forces normalized to the initial stimulus, elicited by 44 subsequent 100 Hz, 500 ms tetanic stimulations separated by 4 seconds. EDL muscles were from 6-week-old WT (black, *n* = 4), ClC-1^–/–^ (blue, *n* = 4), Ca_V_1.1^Δe29^ (orange, *n* = 4), and Ca_V_1.1^Δe29^ ClC-1^–/–^ red, *n* = 4) mice in the (**B**) absence and (**D**) presence of 20 μM verapamil added to the bath for ClC-1^–/–^ (blue, *n* = 4) and Ca_V_1.1^Δe29^ ClC-1^–/–^ (red, *n* = 4) EDLs. Dashed lines in **D** represent the average data presented in **B** as a reference for pretreatment. (**B** and **D**) WT (*n* = 4; female = 2, male = 2), Ca_V_1.1^Δe29^ (*n* = 4; female = 2, male = 2), ClC-1^–/–^ (*n* = 4; female = 2, male = 2), Ca_V_1.1^Δe29^ ClC-1^–/–^ (*n* = 4; female = 2, male = 2). Symbols and closed circles indicate the mean ± SEM. (**B** and **D**) Statistical significance was determined by 2-way ANOVA with Tukey’s post hoc analysis.

The results above indicated that ClC-1^–/–^ mice, regardless of Ca_V_1.1 splicing, showed weakness of hind limb muscles during brief (0.5 s) tetanic stimulation ex vivo, designated here as “fixed weakness.” Previous studies of recessive myotonia in humans and mice have also shown a superimposed transient weakness. This reversible weakness occurs when fibers with genetic loss or pharmacologic block of ClC-1 are rendered temporarily inexcitable (depolarized) by sustained or repetitive muscle activity (17–20). In WT fibers under conditions of ClC-1 blockade, the transient weakness was shown to depend on Ca^2+^ currents through normal (e29^+^) Ca_V_1.1 channels (20). We therefore postulated that e29 skipping and the resulting enhanced Ca^2+^ current may further aggravate transient weakness. We tested this possibility using ex vivo stimulation of the EDL. Muscles from 6-week-old ClC-1^–/–^ and Ca_V_1.1^Δe29^ ClC-1^–/–^ mice were subjected to a series of brief stimuli (0.5 s × 100 Hz) at 4-second intervals for 3 minutes (Figure 3). Representative ClC-1^–/–^ (Figure 3A, blue trace) and Ca_V_1.1^Δe29^ ClC-1^–/–^ EDL (Figure 3A, red trace) force production of the first 15 stimulations, normalized to the maximum force of the first stimulation, demonstrated a transient drop in force production after the first stimulation, which was followed by recovery over subsequent stimulations. The transient weakness in Ca_V_1.1^Δe29^ ClC-1^–/–^ EDLs exhibited an approximately 75% drop in force (Figure 3B, red symbols) compared with approximately 60% in ClC-1^–/–^ EDLs (Figure 3B, blue symbols).

Next, we wanted to determine whether exaggerated transient weakness in Ca_V_1.1^Δe29^ ClC-1^–/–^ muscle depends on Ca^2+^ entry and responds to pharmacologic blockade of Ca_V_1.1. Verapamil is a phenylalkylamine compound in wide clinical use for blocking Ca_V_1.2 channels in cardiac and smooth muscle (21–24). It is also known to block Ca_V_1.1 channels in skeletal muscle (24), but this is not considered clinically relevant because the Ca^2+^ conductance of Ca_V_1.1 is dispensable for ECC of normal skeletal muscle (25). Remarkably, addition of 20 μM verapamil to the bath completely abrogated the transient weakness in ClC-1^–/–^ and Ca_V_1.1^Δe29^ ClC-1^–/–^ EDL muscles ex vivo (Figure 3, C and D, blue and red, respectively). In contrast, addition of verapamil to the bath did not recover the fixed component of weakness in ClC-1^–/–^ or Ca_V_1.1^Δe29^ ClC-1^–/–^ muscles, as shown by non-normalized force plots in Supplemental Figure 6, C and D. Next, we determined the impact of Ca_V_1.1^Δe29^ conductance on transient weakness in the setting of pharmacologic inhibition of ClC-1, where the long-term adaptive response of skeletal muscle to myotonia has not occurred (26). We blocked ClC-1 chloride channels in WT and Ca_V_1.1^Δe29^ EDL muscles with 100 μM 9-anthracene carboxylic acid (9-AC), which is known to block more than 95% of ClC-1 channels in mouse skeletal muscle (27). Notably, this concentration of 9-AC only produced slight transient weakness in WT muscle (Supplemental Figure 7, A and B, blue trace and symbols), which likely reflects the incomplete blockage of Cl^–^ conductance. In contrast, application of 9-AC to Ca_V_1.1^Δe29^ muscle produced severe transient weakness, eliminating 95% of force production, which then recovered to 55% of the original force after 60 seconds before exhibiting the expected muscle fatigue (Supplemental Figure 7, A and B, red trace and symbols). Once again, the addition of verapamil strongly inhibited transient weakness in a dose-dependent manner (Supplemental Figure 7, C–F). Next, we sought to determine whether the observed effect of verapamil on transient weakness was due to blockage of Ca^2+^ entry and not the result of an effect on other ionic currents. To do so, we added Ni^2+^, a nonorganic calcium channel blocker to the muscle bath (28, 29). We found that at 1 mM, extracellular Ni^2+^ virtually eliminated transient weakness in 9-AC–treated WT and Ca_V_1.1^Δe29^ muscle (Supplemental Figure 7, G and H). Taken together with previous work (20), these results confirm that Ca^2+^ currents through Ca_V_1.1 underlie transient weakness in ClC-1–null muscles and show that this effect was enhanced for Ca_V_1.1^Δe29^ channels and rescued by Ca^2+^ channel block.

### Ca_V_1.1^Δe29^ conductance potentiates chloride channel myotonia.

**Figure 4:**
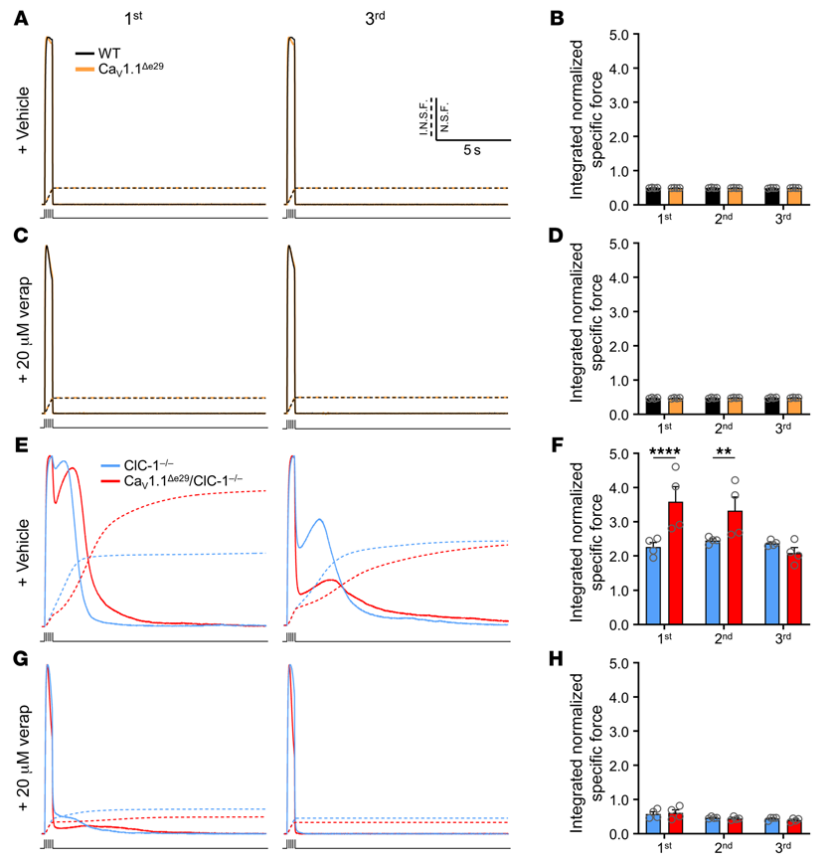
Verapamil significantly reduces myotonia in both Ca_V_1.1^Δe29^ ClC-1^–/–^ and ClC-1^–/–^ mouse muscle. (**A** and **C**) Representative normalized specific force traces of the first (left) and third (right) tetani (150 Hz, 500 ms) from EDL muscles isolated from 6-week-old WT (black) and Ca_V_1.1^Δe29^ (orange) mice in the (**A**) absence and (**C**) presence of 20 μM verapamil added to the bath. Dashed lines represent accumulated force. (**B** and **D**) Average integration of normalized specific force for WT (black) and Ca_V_1.1^Δe29^ (orange) EDL muscles across 3 tetanic stimulations (150 Hz, 500 ms) in the (**B**) absence and (**D**) presence of 20 μM verapamil added to the bath. (**E** and **G**) Representative normalized specific force traces of the first (left) and third (right) tetani (150 Hz, 500 ms) from EDL muscles isolated from 6-week-old ClC-1^–/–^ (blue) and Ca_V_1.1^Δe29^ ClC-1^–/–^ (red) mice in the absence (**E**) and presence (**G**) of 20 μM verapamil added to the bath. Dashed lines represent accumulated force. (**F** and **H**) Average integration of specific force for ClC-1^–/–^ (blue) and Ca_V_1.1^Δe29^ ClC-1^–/–^ (red) EDL muscles across 3 tetanic stimulations (150 Hz, 500 ms) in the (**B**) absence and (**D**) presence of 20 μM verapamil added to the bath. (**B**, **D**, **F**, and **H**) WT (*n* = 4; female = 2, male = 2), Ca_V_1.1^Δe29^ (*n* = 4; female = 2, male = 2), ClC-1^–/–^ (*n* = 4; female = 2, male = 2), Ca_V_1.1^Δe29^ ClC-1^–/–^ (*n* = 4; female = 2, male = 2). All specific force traces were normalized to the peak specific force. Symbols and open circles represent individual mice; bars indicate the mean ± SEM. Notes: Contralateral EDL muscles were used for each untreated and treated experiment. All traces were plotted with the same scale of time and normalized force for comparison. The same traces without force normalization are shown in Supplemental Figure 8. (**B**, **D**, **F**, and **H**) **P < 0.01 and ****P < 0.0001, by 1-way ANOVA with Tukey’s post hoc analysis.

Given the impact of Ca_V_1.1^Δe29^ channels on transient weakness and the interplay of transient weakness and myotonia, we also wanted to determine the effects of Ca_V_1.1^Δe29^ on myotonia in ClC-1 deficient fibers. Previous work demonstrated that Ca^2+^ currents through Ca_V_1.1 (+e29) contribute sequentially to myotonia and transient weakness (30). Using EDL muscles isolated from 6-week-old mice, myotonia was taken as the inappropriate continuation of force production after tetanic stimulation was stopped (Figure 4). We elicited muscle contraction using 3 successive rounds of tetanic stimulation (150 Hz, 500 ms), each separated by 3 minutes of rest. This rest period reduces the well-known “warm-up” phenomenon of chloride channel myotonia (15), in which myotonia wanes with repeated bouts of muscle activation. The force traces were normalized to peak force and then integrated over time to quantify myotonia (dashed lines in Figure 4, A, C, E, and G). The integrated signals for the first, second, and third stimulations were then compared (Figure 4, B, D, F, and H). As expected, we observed no myotonia in WT (black) or Ca_V_1.1^Δe29^ (orange) muscles (Figure 4, A and B). In contrast, ClC-1^–/–^ (blue) and Ca_V_1.1^Δe29^ ClC-1^–/–^ (red) muscles both showed robust myotonia, but the severity was greater in bi-channelopathy muscle (Figure 4, E and F). Interestingly, despite the 3-minute rest interval, the myotonia in Ca_V_1.1^Δe29^ ClC-1^–/–^, but not in ClC-1^–/–^, muscle demonstrated warm-up, showing a 42% decrease over successive stimulations (Figure 4F, red), without any corresponding drop in peak specific force (Supplemental Figure 8). We examined contralateral muscles in the presence of 20 μM verapamil. Remarkably, we observed that the myotonic after-contractions were virtually eliminated by verapamil in both ClC-1^–/–^ (blue) and Ca_V_1.1^Δe29^ ClC-1^–/–^ (red) EDL muscles (Figure 4, G and H, and Supplemental Figure 10B). We also compared the effect of Ca_V_1.1^Δe29^ channels on myotonia brought about by acute pharmacologic blockade of ClC-1 channels (Supplemental Figure 9). As with our studies of transient weakness, we observed differences between genetic and pharmacologic myotonia in both WT and Ca_V_1.1^Δe29^ muscles. Comparing the integrated normalized force production, myotonia was significantly less severe in 9-AC–treated WT and Ca_V_1.1^Δe29^ muscles (Figure 4F) as compared with the genetic counterparts (ClC-1^–/–^ and Ca_V_1.1^Δe29^ ClC-1^–/–^) (Supplemental Figure 9E; see comparative analysis in Supplemental Figure 10). Unlike Ca_V_1.1^Δe29^ ClC-1^–/–^ muscles, there was no indication of warm-up in 9-AC–treated Ca_V_1.1^Δe29^ muscle from the first to the third stimulus series (Supplemental Figure 9E). Furthermore, we observed no waxing or waning of post-stimulation myotonia in 9-AC–treated WT or Ca_V_1.1^Δe29^ EDL muscles (Supplemental Figure 9E), in contrast to what was observed in all force recordings from ClC-1^–/–^ and Ca_V_1.1^Δe29^ ClC-1^–/–^ EDL muscles (Figure 4E). Regardless of these differences, application of 5 and 20 μM verapamil again showed a dose-dependent reduction of 9-AC–induced myotonia in both WT and Ca_V_1.1^Δe29^ EDLs (Supplemental Figure 9, F and G), without a significant impact on peak force production (Supplemental Figure 11). Again, we wanted to determine whether the effects of verapamil on myotonia were due to blockage of Ca^2+^ entry through Ca_V_1.1 channels. To do so, we added 1 mM Ni^2+^ to the bath and observed a significant reduction in myotonia in 9-AC–treated WT and Ca_V_1.1^Δe29^ muscles (Supplemental Figure 9H) that was comparable to the addition of 5 μM verapamil (Supplemental Figure 9F). Importantly, the application of verapamil or nickel did not affect the tetanic contraction properties of WT or Ca_V_1.1^Δe29^ EDL muscles in the absence of myotonia (Supplemental Figures 9, A–D, and Supplemental Figure 11, A–D). Taken together, these results indicate that Ca^2+^ conductance potentiated myotonia in the context of normal Ca_V_1.1^+e29^ channels. This effect was further aggravated when Ca_V_1.1 exon 29 was skipped, and in both circumstances, the myotonia was mitigated by verapamil blockage of Ca_V_1.1 conductance. It was also apparent that Ca_V_1.1^Δe29^ unmasked myotonia even when loss of ClC-1 conductance (pharmacologic block) was incomplete and that increased Ca^2+^ conductance through Ca_V_1.1^Δe29^ enhanced the warm-up phenomenon.

### Long-term verapamil administration improves survival, body weight, and motility of Ca_V_1.1^Δe29^ ClC-1^–/–^ mice.

**Figure 5:**
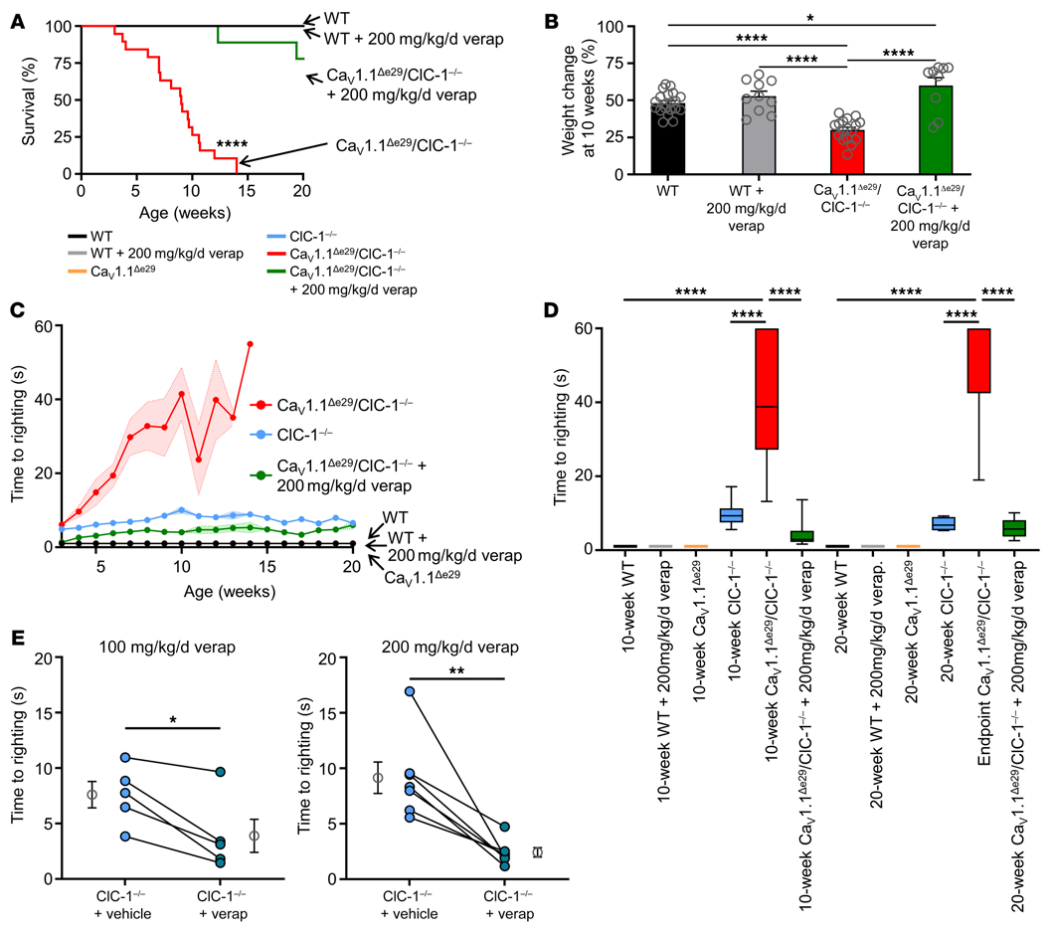
Verapamil treatment improves survival, body weight, and motility of Ca_V_1.1^Δe29^ ClC-1^–/–^ mice. (**A**) Kaplan-Meier survival analysis of WT mice (*n* = 10; female = 5, male = 5), WT mice treated with 200 mg/kg/d verapamil (verap) (*n* = 10; female = 5, male = 5), Ca_V_1.1^Δe29^ ClC-1^–/–^ mice (*n* = 19; female = 9, male = 10), and Ca_V_1.1^Δe29^ ClC-1^–/–^ mice treated with verapamil (*n* = 9; female = 5, male = 4). Verapamil was dosed in mouse nutrition/hydration food cups. Note: log-rank analysis of Ca_V_1.1^Δe29^ ClC-1^–/–^ versus WT mice, WT mice treated with 200 mg/kg/d verapamil, and Ca_V_1.1^Δe29^ ClC-1^–/–^ mice treated with 200 mg/kg/d verapamil; *P* < 0.0001, *P* < 0.0001, and *P* < 0.0001, respectively. (**B**) Percentage of body weight change from weaning at 10 weeks in WT mice (*n* = 20; female = 10, male = 10), WT mice treated with 200 mg/kg/d verapamil (*n* = 10; female = 5, male = 5), Ca_V_1.1^Δe29^ ClC-1^–/–^ mice (*n* = 35; female = 14, male = 21), and Ca_V_1.1^Δe29^ ClC-1^–/–^ mice treated with verapamil (*n* = 9; female = 5, male = 4). (**C**) Weekly time of righting reflex analysis of Ca_V_1.1^Δe29^ ClC-1^–/–^ mice (red), ClC-1^–/–^ mice (light blue), Ca_V_1.1^Δe29^ mice (orange), WT mice (black), Ca_V_1.1^Δe29^ ClC-1^–/–^ mice treated with verapamil (green), and WT mice treated with verapamil (gray). (**D**) Average time of righting reflex in vehicle- and verapamil-treated mice at 10 and 20 weeks of age. Note: Untreated Ca_V_1.1^Δe29^ ClC-1^–/–^ mice did not survive to 20 weeks of age, therefore the last recording before death was documented. Box indicates Q1, the median, and Q3; whiskers show the minimum and maximum. (**C** and **D**) Ten- and 20-week-old WT mice (*n* = 10; female = 5, male = 5), WT mice treated with 200 mg/kg/d verapamil (*n* = 10; female = 5, male = 5), Ca_V_1.1^Δe29^ mice (*n* = 8; female = 4, male = 4), ClC-1^–/–^ mice (*n* = 19; female = 9, male = 10), Ca_V_1.1^Δe29^ ClC-1^–/–^ mice (*n* = 15; female = 7, male = 8), Ca_V_1.1^Δe29^ ClC-1^–/–^ mice treated with verapamil (10-week-old mice, *n* = 16; female = 7, male = 9), Ca_V_1.1^Δe29^ ClC-1^–/–^ mice treated with verapamil (20-week-old mice, *n* = 7; female = 4, male = 3). (**E**) Paired before (light circles) and after (dark circles) 2 weeks of verapamil treatment of ClC-1^–/–^ mice at 100 mg/kg/d (left; *n* = 5; female = 3, male = 2) and 200 mg/kg/d (right; *n* = 6; female = 3, male = 3) dosing in nutrition/hydration food cups. Symbols and closed circles indicate individual mice; open circles indicate the mean ± SEM. *P < 0.05, **P < 0.01, and ****P < 0.0001, by log-rank analysis (**A**); 1-way ANOVA (**B** and **D**) and 2-way ANOVA (**C**) with Tukey’s post hoc analysis; and paired *t* test (**E**).

Since short-term exposure to verapamil inhibited transient weakness and myotonia ex vivo in Ca_V_1.1^Δe29^ ClC-1^–/–^ muscles, we sought to determine whether long-term administration in vivo would improve survival and muscle function in bi-channelopathy mice. Previous work has shown that oral administration of verapamil mixed in food at an estimated dose of 100 mg/kg/d was well tolerated over 16 weeks in mice lacking the sarcoglycan-sarcospan complex in smooth muscle and rescued the cardiomyopathy caused by microvascular defects (31). In a pilot study of oral feeding, we found that estimated regimens of 100 and 200 mg/kg/d verapamil, starting at the time of weaning (3 weeks of age), were well tolerated in Ca_V_1.1^Δe29^ ClC-1^–/–^ mice and appeared to extend survival (*n* = 3 and *n* = 4, respectively; Supplemental Figure 12). Since 3 of 4 mice on 200 mg/kg/d survived to 20 weeks of age, we carried this regimen forward in a repeat study of a larger cohort. Once again, we found that verapamil significantly (*P* ≤ 0.0001) extended the survival of Ca_V_1.1^Δe29^ ClC-1^–/–^ mice. Eight of 9 mice survived beyond 14 weeks of age, which was never observed in the untreated mice receiving the same diet, and 7 of 9 mice survived to the age of 20 weeks (Figure 5A, green line) when they were euthanized for analysis of muscle function and histology. Verapamil-treated Ca_V_1.1^Δe29^ ClC-1^–/–^ mice showed significant weight gain that was equivalent to that of the verapamil-treated WT mice (Figure 5B).

To assess the effect of verapamil on general mobility in Ca_V_1.1^Δe29^ ClC-1^–/–^ mice, we again utilized the righting response. Chronic administration of verapamil significantly (*P* ≤ 0.0047) improved the righting response of Ca_V_1.1^Δe29^ ClC-1^–/–^ mice (Figure 5C, green symbols) to near-WT levels (Figure 5C, black symbols). The average righting times for 10- and 20-week-old mice (or the last TRR trial before death) are presented in Figure 5D. Surprisingly, Ca_V_1.1^Δe29^ ClC-1^–/–^ mice treated with verapamil showed a modest improvement of the righting response over untreated ClC-1^–/–^ mice. Since acute exposure to verapamil had also reduced transient weakness and myotonia in single-channelopathy ClC-1^–/–^ muscle and 9-AC–treated WT muscle ex vivo (see above), in the context of WT (+e29) Ca_V_1.1, we also examined ClC-1^–/–^ mice before and after 2 weeks of 100 and 200 mg/kg/d verapamil administration by oral feeding. The righting response improved at both doses (Figure 5E), with 200 mg/kg/d treatment having the greater effect.

### Long-term verapamil administration improves respiratory and diaphragm function in Ca_V_1.1^Δe29^ ClC-1^–/–^ mice.

**Figure 6:**
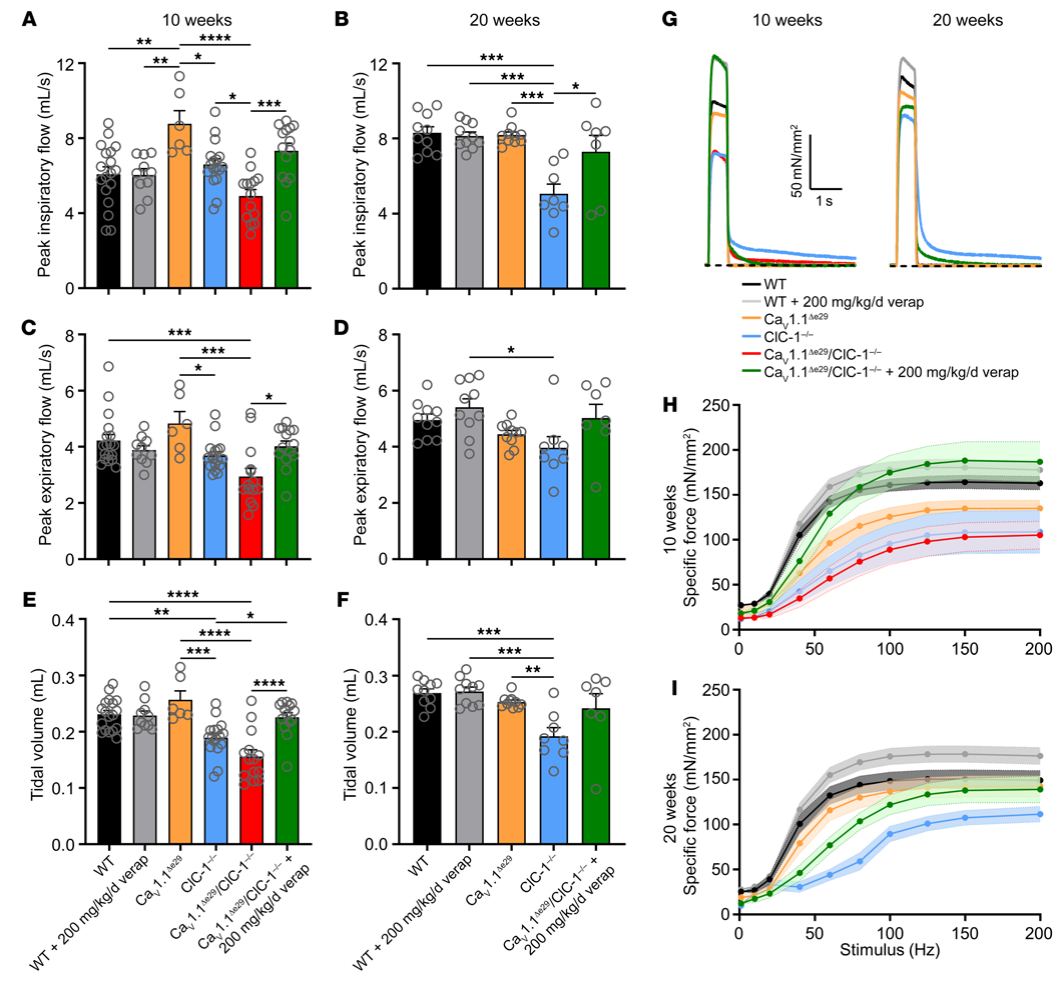
Verapamil treatment significantly improves respiratory function and diaphragm strength in Ca_V_1.1^Δe29^ ClC-1^–/–^ mice. WBP for (**A**, **C**, and **E**) 10-week-old WT mice (*n* = 18; female = 9, male = 9), WT mice treated with 200 mg/kg/d verapamil (*n* = 10; female = 5, male = 5), Ca_V_1.1^Δe29^ mice (*n* = 6; female = 3, male = 3), ClC-1^–/–^ mice (*n* = 17; female = 8, male = 9), Ca_V_1.1^Δe29^ ClC-1^–/–^ mice (*n* = 14; female = 7, male = 7), and Ca_V_1.1^Δe29^ ClC-1^–/–^ mice treated with verapamil (*n* = 14, female = 7, male = 7) and (**B, D** and **F**) 20-week-old WT mice (*n* = 10; female = 5, male = 5), WT mice treated with 200 mg/kg/d verapamil (*n* = 10; female = 5, male = 5), Ca_V_1.1^Δe29^ mice (*n* = 10; female = 4, male = 6), ClC-1^–/–^ mice (*n* = 8; female = 4, male = 4), Ca_V_1.1^Δe29^ ClC-1^–/–^ mice treated with verapamil (*n* = 7; female = 4, male = 3). (**A** and **B**) PIFR (mL/s) and (**C** and **D**) PEFR (mL/s) of respiration. (**E** and **F**) Tidal volume (mL) of respiration. (**G**) Representative tetanic (150 Hz, 500 ms) specific force traces from diaphragm strips isolated from 10-week-old (left) and 20-week-old (right) mice of the indicated genotypes and treatment groups. (**H** and **I**) Plot of the average stimulation frequency dependence of specific force generated from diaphragm strips isolated from (**H**) 10-week-old (*n* values indicate individual EDL muscles) WT mice (*n* = 10; female = 5, male = 5), WT mice treated with 200 mg/kg/d verapamil (*n* = 6; female = 3, male = 3), Ca_V_1.1^Δe29^ mice (*n* = 5; female = 3, male = 2), ClC-1^–/–^ mice (*n* = 5; female = 2, male = 3), Ca_V_1.1^Δe29^ ClC-1^–/–^ mice (*n* = 7; female = 4, male = 3), and Ca_V_1.1^Δe29^ ClC-1^–/–^ mice treated with verapamil (*n* = 7; female = 3, male = 4)) and (**I**) 20-week-old (*n* values indicate individual EDL muscles) WT mice (*n* = 8; female = 4, male = 4), WT mice treated with 200 mg/kg/d verapamil (*n* = 8; female = 4, male = 4), Ca_V_1.1^Δe29^ mice (*n* = 7; female = 4, male = 3), ClC-1^–/–^ mice (*n* = 7; female = 3, male = 4), and Ca_V_1.1^Δe29^ ClC-1^–/–^ mice treated with verapamil (*n* = 7; female = 4, male = 3). Symbols and open circles represent individual mice; bars and closed circles indicate the mean ± SEM. Note: Untreated Ca_V_1.1^Δe29^ ClC-1^–/–^ mice did not survive to 20 weeks of age. *P < 0.05, **P < 0.01, ***P < 0.001, and ****P < 0.0001, by 1-way ANOVA (**A**–**F**) and 2-way ANOVA (**H** and **I**) with Tukey’s post hoc analysis.

Respiratory failure is a common complication of DM1 in its later stages (32). We therefore used whole-body plethysmography (WBP) to assess respiratory function in bi-channelopathy mice. As compared with WT controls, the peak inspiratory flow rate (PIFR), peak expiratory flow rate (PEFR), and tidal volume (TV) were all reduced in Ca_V_1.1^Δe29^ ClC-1^–/–^ mice at 10 weeks of age (Figure 6, A, C, and E). Long-term administration of verapamil rescued the deficits of PIFR, PEFR, and TV to values similar to those for WT mice at ages 10 and 20 weeks (Figure 6, B, D, and F). Since diaphragm muscle is preferentially affected in DM1, we investigated whether respiratory impairment in Ca_V_1.1^Δe29^ ClC-1^–/–^ mice is accompanied by reduced diaphragm function. We examined the force-frequency relationship using strips of diaphragm muscle isolated from 10-week-old Ca_V_1.1^Δe29^ ClC-1^–/–^ mice and found a marked reduction of tetanic force compared with WT mice at frequencies greater than 20 Hz (Figure 6H). Remarkably, the deficit of diaphragmatic force was also rescued by chronic administration of verapamil (Figure 6H), even though verapamil was not added to the bath during the force measurements. This correction of diaphragm force production was maintained at 20 weeks of age in Ca_V_1.1^Δe29^ ClC-1^–/–^ mice, with peak tetanic force production similar to that for WT mice (Figure 6I). Representative tetanic force traces from 10- and 20-week-old mice (Figure 6G) demonstrated no overt difference in shortening velocity between the groups, however relaxation was markedly prolonged in diaphragm muscle isolated from verapamil-treated Ca_V_1.1^Δe29^ ClC-1^–/–^ mice, which verified that the anti-myotonia effects were “washed out” during the force measurements, further supporting a myoprotective effect of chronic exposure to verapamil in vivo. Taken together, the rescue of diaphragm muscle function by verapamil supports the observed improvement of respiratory function. We next wanted to determine whether verapamil had myoprotective effects on limb muscles. Indeed, we found that EDL muscles isolated from Ca_V_1.1^Δe29^ ClC-1^–/–^ mice that received long-term administration of 200 mg/kg/d verapamil had significantly (*P* ≤ 0.0001) increased specific force production upon tetanic stimulations at 10 weeks (Supplemental Figure 13, A and B), and this was sustained at 20 weeks of age (Supplemental Figure 13, C and D). Furthermore, the muscle weight and cross-sectional area (CSA) of dissected 10-week-old Ca_V_1.1^Δe29^ ClC-1^–/–^ EDL muscles were significantly smaller than those of age-matched WT mice (Supplemental Figure 13E), suggesting that the muscles were atrophic. Verapamil-treated Ca_V_1.1^Δe29^ ClC-1^–/–^ mice showed a significant increase in weight and CSA of isolated EDL muscles at 10 weeks that did not differ from age-matched WT mice at 10 weeks (Supplemental Figure 13E) or 20 weeks (Supplemental Figure 13F).

## Discussion

ClC-1 and Ca_V_1.1 channels are both required for normal functioning of skeletal muscle, and both undergo perinatal transitions in isoform expression. The predominant ClC-1 isoform expressed in fetal muscle includes a cryptic exon 7a, causing a shift of reading frame, nonsense-mediated decay of mRNA, truncation of channel protein, and loss of ion channel activity (2, 11). As fibers develop and the transverse tubule system matures, the alternative splicing shifts to exclude exon 7a under the influence of MBNL, leading to high levels of chloride conductance. The chloride conductance then acts as an electrical buffer to stabilize the transmembrane potential during muscle activity (33–35). In recessive myotonia congenita (RMC), loss of ClC-1 is known to cause depolarization of the surface membrane during muscle activity, involuntary runs of action potentials (myotonia), and reversible loss of excitability (transient weakness).

Ca_V_1.1 is the voltage sensor for ECC. Upon muscle excitation, Ca_V_1.1 undergoes a conformation change that triggers Ca^2+^ release from intracellular stores through physical coupling to RyR1. In fetal muscle, exon 29 is skipped in approximately half the Ca_V_1.1 mRNAs, encoding Ca_V_1.1^Δe29^ protein that has substantial Ca^2+^ conductance (12, 36). The postnatal splicing of Ca_V_1.1 shifts to nearly 100% production of Ca_V_1.1^+e29^, again driven by MBNL, which has much lower Ca^2+^ conductance (37). Interestingly, the Ca^2+^ conductance of Ca_V_1.1^+e29^ is dispensable for ECC of mature muscle (25) but does contribute to fiber depolarization and transient weakness when ClC-1 is absent (20). Furthermore, when the switch to Ca_V_1.1^+e29^ is entirely prevented by homozygous deletion of e29 in mice, the constitutive expression of Ca_V_1.1^–e29^ leads to changes in fiber type specification and age-dependent signs of mitochondrial toxicity (9).

The CTG expansion in DM1 is highly unstable in somatic cells, causing age-dependent growth of the expanded repeat at different rates in different tissues. When size of the repeat and extent of toxic RNA reaches a threshold level in skeletal muscle, splicing of ClC-1 and Ca_V_1.1 reverts to fetal isoforms (36), bringing the circumstance of chloride channel myotonia together with increased Ca^2+^ entry through Ca_V_1.1^Δe29^. The main finding of our study is that combined Ca_V_1.1 ClC-1 bi-channelopathy is highly deleterious, causing aggravated myotonia and transient weakness, fixed weakness, respiratory impairment, and a marked reduction of lifespan. It remains possible, if not likely, that other ECC-related splicing defects, such as expression of RyR1^–e70^ and SERCA1^–e22^, may also contribute, but our results suggest a central role for Ca_V_1.1/ClC-1 bi-channelopathy in the functional impairment of muscle.

Extrapolation of our findings to humans is not straightforward for several reasons, including the selective patterns of muscle involvement in DM1, effects of DM1 on the expression of other genes, and limited survival of bi-channelopathy mice. It is important to note that other splicing defects, including those in *BIN1* and *DYS*, have been previously implicated in DM1 myopathy (8, 38). It is therefore clear that Cl^–^/Ca^2+^ bi-channelopathy does not provide a unitary explanation for DM1 myopathy. To further dissect the contributory role of Cl^–^/Ca^2+^ bi-channelopathy, one approach in future studies is to subtract the influence of Ca_V_1.1^Δe29^ from the overall DM1 milieu using Ca^2+^ blockers. However, this awaits the development of authentic models with longer repeats that fully replicate the RNA toxicity observed in patients with DM1. Another important difference is that bi-channelopathy in our model is constitutive throughout all skeletal muscles, whereas DM1 has selective effects on particular muscles. For example, the myotonia in DM1 is often quite severe in distal limb and oromandibular muscles, which now can be attributed to combined ClC-1 loss and Ca_V_1.1 e29 skipping in those muscles, whereas in proximal limb muscles, the myotonia and weakness are much less apparent. The mechanisms underlying selective muscle involvement are currently unknown, but evidence supports the idea that the extent of splicing dysregulation is associated with the degree of muscle weakness within and between patients (10). Therefore, it is reasonable to expect that observations from bi-channelopathy mice may apply most directly to DM1 muscles that exhibit a near-complete loss of ClC-1 and levels of Ca_V_1.1 e29 skipping approaching or exceeding heterozygous Ca_V_1.1^Δe29^ mice, and this clearly does occur in distal limb muscles that are preferentially affected, such as tibialis anterior (10). Finally, the duration of Cl^–^/Ca^2+^ channelopathy was limited by the short survival of bi-channelopathy mice in this study. Therefore, in future studies, it will be important to examine the effects of Cl^–^/Ca^2+^ bi-channelopathy over longer intervals in specific muscle groups using conditional Ca_V_1.1^Δe29^ mice. A limitation of our study is that the cause of death in bi-channelopathy mice was not clearly defined. Almost certainly, the cause of death originates from skeletal muscle, since ClC-1 and Ca_V_1.1 are both expressed almost exclusively in this tissue. Since the bi-channelopathy mice showed reduced weight gain and diaphragmatic weakness, it is likely that both inanition and respiratory insufficiency may be contributing factors.

Our results indicate a complex physiologic interplay of Cl^–^ and Ca^2+^ channels, in which increased Ca^2+^ entry through Ca_V_1.1^Δe29^ aggravated the myotonia and transient weakness from Cl^–^ channelopathy and, conversely, in which Cl^–^ channel myotonia may have aggravated the myopathy caused by excessive Ca^2+^ entry through Ca_V_1.1^Δe29^ channels. To a surprising extent, it appears that this chain of events can be broken by a single intervention at the level of Ca^2+^ channels. Verapamil blockade of Ca_V_1.1 showed striking benefits for survival and respiratory function in bi-channelopathy mice and also improved the myotonia and transient weakness in bi-channelopathy muscle fibers. In fact, the latter effects were even observed in single-channelopathy ClC-1^–/–^ fibers. Whether verapamil can alleviate the long-term mitochondrial toxicity in single- or bi-channelopathy Ca_V_1.1^Δe29^ mice remains to be determined. Interestingly, Grant and colleagues reported in 1987 that another Ca^2+^ channel blocker, nifedipine, had anti-myotonia effects in DM1 (39). To our knowledge, confirmatory studies were never carried out, possibly because genes for nondystrophic myotonia were discovered soon thereafter and shown to encode sodium or Cl^–^ channels, undermining a potential connection to Ca^2+^ channels.

We caution against a direct application of our results to the treatment of patients with DM1. Bi-channelopathy mice are not expected to exhibit any of the cardiac features of DM1, such as disease of the conduction system, which is potentially exacerbated by Ca^2+^ channel blockers. Further work is needed to identify agents that benefit Ca_V_1.1^Δe29^ channelopathy without aggravating conduction system disease or the tendency for low blood pressure in DM1. This includes the possibility of developing agents that are selective for Ca_V_1.1 channels in skeletal muscle over Ca_V_1.2 channels in cardiac and smooth muscle, which to our knowledge has not been a priority previously.

## Methods

### Generation of mouse lines.

CRISPR/Cas9 was used to generate forced exon deletion mice. Exon 29 of *Cacna1s*, exon 22 of *Serca1*, and exon 70 of *Ryr1* were removed by targeting approximately 50 nucleotides into the intronic sequence on either side of the intron/exon border to ensure that both the exon and the splice donor/acceptor sequence were removed. Founder mouse lines were crossed with C57BL/6J mice (The Jackson Laboratory) for more than 6 generations to eliminate possible off-target mutations. sgRNA sequences used to generate mice are listed in Supplemental Table 2. The removal of exon 29 in *Cacna1s*, exon 22 in *Serca1*, and exon 70 in *Ryr1* was validated by RNA isolation of tibialis anterior and Sanger sequencing of RT-PCR products between exons noted for each transcript in Supplemental Table 3. The primers used for Sanger sequencing are also noted in Supplemental Table 3. To generate Ca_V_1.1^Δe29^ ClC-1^–/–^, SERCA1^Δe22^ ClC-1^–/–^, and RyR1^Δe70^ ClC-1^–/–^ mice, *adr-mto2J* mice (The Jackson Laboratory), which have a frameshift mutation in *Clcn1* (ClC-1^–/–^) causing a recessive generalized chloride channel myotonia, were bred with Ca_V_1.1^Δe29/Δe29^, SERCA1^Δe22/Δe22^, and RyR1^Δe70/Δe70^ mice (11). *Adr-mto2J* mice were obtained from The Jackson Laboratory and bred to achieve congenic C57BL/6J strains. WT C57BL/6J control mice were obtained from The Jackson Laboratory when littermates could not be used. For Ca_V_1.1^Δe29^ ClC-1^–/–^ mice, terminal endpoints were determined by the age of the mouse or when the righting reflex time was greater than 60 seconds.

### Verapamil administration.

Mice were provided verapamil via ingestion of Nutra-Gel Complete Nutrition food (Bio-Serv) to a final dose of approximately 200 mg/kg/d verapamil [V4629, (±)-verapamil hydrochloride, MilliporeSigma]. Food cups contained 0.1% w/w verapamil, which was first solubilized in 1 mL dH_2_O. Mice that did not receive verapamil, were provided Nutra-Gel Complete Nutrition food that had 1 mL dH_2_O added to serve as a vehicle control. Mice were provided food cups immediately after weaning, and food was changed daily and weighed for consumption. No other food source was provided. Water bottles were provided and changed weekly.

### Body weight measurements.

Body weight of the mice was measured every other day following weaning. For statistical analysis, 2-way ANOVA was performed on the average of each week’s measurements. The body weight change percentage at 10 weeks was determined, with the first weight measurement taken after weaning and at 10 weeks of age. For these data, a 1-way ANOVA with multiple comparisons was performed.

### Time of righting reflex measurements.

The time of righting reflex was tracked weekly after the start of verapamil treatment. Mice were held in a supine position on a level surface and then released. Two investigators blinded to genotype and treatment began timing immediately after release, and timing was stopped once all 4 paws were down on the surface. This was repeated for a total of 10 timed righting responses with a minimum of 5 minutes in between each recording to avoid the warm-up phenomenon (15). Recordings of less than 1 second were recorded as “1,” and righting was considered a failure if righting took more than 60 seconds. If failure occurred, no further recordings were measured for that mouse for that week. The 2 longest and 2 shortest recordings for each mouse were removed, and the average of the middle 6 recordings was used for analysis. We performed 2-way ANOVA on the week-to-week results and 1-way ANOVA with multiple comparisons for the time point data (10-week and 20-week).

### WBP.

We performed WBP (Buxco Small Animal Whole Body Plethysmography, Data Science International) to measure respiratory function at 10 weeks (all groups) and 20 weeks (excluding untreated Ca_V_1.1^Δe29^ ClC-1^–/–^). Mice were acclimated to the system for 2 days, with data collected on day 3. For all 3 days, a single mouse was placed conscious and unrestrained in the WBP chamber and subjected to a 15-minute acclimation period, followed by 10 minutes of data acquisition (40). Average data from the 10-minute acquisition period were analyzed by 1-way ANOVA with multiple comparisons.

### Whole-cell patch-clamp recording of calcium currents from dissociated adult FDB fibers.

Mice at approximately 4 weeks postnatal age were sacrificed by cervical dislocation and decapitation. Isolated FDB muscle fibers for electrophysiology experiments were obtained by previously described methods (4). Briefly, FDB muscles were immediately microdissected and submerged in standard electrophysiology Ringer’s solution (146 mM NaCl, 5 mM KCl, 2 mM CaCl_2_, 1 mM MgCl_2_, and 10 mM HEPES, pH 7.4, with NaOH). Excised muscles were then digested for 1 hour in an oscillating water bath at 37°C in Ringer’s solution supplemented with 2 mg/mL collagenase A (Roche). Following enzymatic digestion, the muscles were mechanically dissociated by trituration with fire-polished Pasteur pipettes of decreasing bore diameter to obtain isolated single FDB fibers. Fibers were then plated on 35 mm plastic cell culture dishes for experiments. All patch-clamp experiments were completed within 8 hours of the sacrifice of the mouse. To record Ca_V_1.1 calcium currents with the whole-cell patch-clamp, solutions were designed to isolate calcium currents. Tetrodotoxin (TTX) (Cayman Chemical) was used to block sodium currents; tetraethylammonium (TEA), 4-aminopyridine (4-AP), and cesium were used to block potassium currents; and 9-AC was used to block ClC-1 Cl^–^ currents. *N*-benzyl-*p*-toluene sulphonamide (BTS) was used to inhibit contraction. The external recording solution consisted of 150 mM TEA_2_-methanesulfonate, 10 mM Ca-methanesulfonate, 10 mM HEPES, 2 mM MgCl_2_, 5 mM 4-AP, 0.1 mM 9-AC, 0.1 mM BTS, and 0.001 mM tetrodotoxin, adjusted to pH 7.4 with TEA-OH. Low-resistance pipettes were fashioned from thin-walled borosilicate glass with a Sutter P-97 puller (Sutter Instruments) and fire polished to a resistance of less than 1 MΩ. Pipettes were filled with an internal solution consisting of 145 mM Cs-aspartate, 15 mM Cs_2_-EGTA, 10 mM MgATP, 1.5 mM CaCl_2_, and 10 mM HEPES, adjusted to pH 7.4 with CsOH. Currents were recorded with standard voltage-step protocols with 500 ms voltage steps from –50 to –80 to 80 mV at 10 mV intervals, as described previously (41). Data were collected using an Axopatch 200B amplifier (Axon Instruments) and digitized with a Digidata 1550b (Axon Instruments). A –P/4 online subtraction protocol was used to correct linear components of leak and capacitive currents. To limit voltage error, whole-cell parameters were adjusted, and series resistance was compensated (90%). Recordings were sampled at 10 kHz and filtered at 2 kHz. Cell capacitance and other passive properties of the muscle fibers were either obtained via Clampex 10 (Molecular Devices) or calculated on the basis of the integration of a 10 mV voltage step from resting. Currents were normalized to fiber capacitance to obtain current densities (pA/pF). All experiments were completed at room temperature. Peak current densities obtained for the family of voltage steps were plotted and fit using the following equation to obtain I–V curves: *I* = *G*max × (*V*−*V*rev)/(1+exp[−(*V*−*V*1/2)/*kG*]), where *I* corresponds to the peak current of a specific test potential normalized to cell capacitance, *G*max is the maximum calcium conductance, *V*1/2 is the half-maximal activation potential, *V*rev is the reversal potential, and *kG* is the slope factor.

### Histology and fiber typing.

For H&E staining as well as fiber typing by immunohistochemistry, the tibialis anterior and diaphragm were isolated from 10- and 20-week-old mice (*n* = 5 mice/group) and snap frozen. Transverse 10 μm cryosections from each muscle were collected onto glass slides. Slides were submerged in hematoxylin for 3.5 minutes, followed by 1 wash in ddH_2_O, 1% HCl, ddH_2_O, ammonia water, and ddH_2_O and then submerged in eosin for 5 minutes. Slides were dehydrated by two 30-second incubations in 95% ethanol, two 30-second incubations in 100% ethanol, and one 30-second incubation followed by a 5-minute incubation in Hemo-De (xylene substitute). The slides were then mounted with Permount (Thermo Fisher Scientific) and imaged on an Olympus BX53. Immunohistochemistry was used to determine fiber type, as described by Bachman et al. (42). Briefly, slides were permeabilized once with PBST (PBS with 0.2% Triton X-100) for 10 minutes and then blocked for 30 minutes in 10% normal goat serum (NGS) (Gibco, Thermo Fisher Scientific) in 1× PBS at room temperature. After this, the slides were blocked for 60 minutes in 3% AffiniPure Fab fragment anti–mouse IgG (H&L) (Jackson Immunoresearch) (0.1 mg/mL) with 2% NGS in 1× PBS at room temperature, and then washed 3 times for 10 minutes each in PBS. Slides were incubated for 14–18 hours at 4°C in primary antibodies (noted in Supplemental Table 4). Next, slides were washed 3 times for 10 minutes with 1× PBS at room temperature, and then secondary antibodies (see Supplemental Table 4) were added and incubated for 60 minutes. After another set of 3 washes for 10 minutes in 1× PBS at room temperature, the slides were mounted with Fluoromount-G (Invitrogen, Thermo Fisher Scientific). The slides were imaged using a Keyence BZ-X810 All-in-One Florescence Microscope (Leica).

### Ex vivo muscle contraction.

Muscle strength and frequency dependence were measured by ex vivo muscle contraction performed on an Aurora Scientific 1200A ex vivo system equipped with an 809B muscle testing system with a 300C-LR force transducer and a 701C stimulator (Aurora Scientific). For this experiment, 10- and 20-week-old WT mice, WT mice treated with verapamil, Ca_V_1.1^Δe29^ mice, ClC-1^–/–^ mice, untreated Ca_V_1.1^Δe29^ ClC-1^–/–^ mice (only at 10 weeks of age), and Ca_V_1.1^Δe29^ ClC-1^–/–^ plus verapamil-treated mice were used. Mice were anesthetized by 2% inhaled isoflurane. For the EDL muscle, the proximal and distal tendons of the EDL were exposed after removal of the tibialis anterior and tied using suture thread. The proximal tendon was set on an immobile post, and the distal tendon was hooked to the force transducer. For the diaphragm, a 4 mm strip was dissected from the right costal hemidiaphragm as described in Hakim et al. (43). A suture was used to connect the diaphragm strip to the stationary post from the ribs, and the central tendon was secured to the force transducer. For both muscle groups, the muscles were submerged between platinum electrodes in warmed (30°C) and oxygenated (95% O_2_ and 5% CO_2_) Ringer’s buffer (1.2 mM NaH_2_PO_4_, 1 mM MgSO_4_, 4.83 mM KCl, 137 mM NaCl, 2 mM CaCl_2_, 10 mM glucose, 24 mM NaHCO_3_ at pH 7.4) (44). EDL and diaphragm muscles were equilibrated for 10 minutes before determination of the optimal length (L_o_) and supramaximal output (120% stimulating voltage) (45). Muscles were then subjected to a twitch warm-up protocol (3 times 0.2 ms, separated by 20 s) and a tetanus warm-up protocol (3 times 500 ms trains of 0.2 ms pulses, delivered at 150 Hz, separated by 1 min) before frequency dependence was determined was determined (in all stimulation paradigms, the stimulation duration was 0.2 ms). Frequency dependence was determined with increasing 500 ms stimulations separated by 1 minute. For EDL muscles, the stimulations were as follows: 1 Hz (200 ms), 25 Hz (500 ms onward), 50 Hz, 75 Hz, 100 Hz, 125 Hz, 150 Hz, 175 Hz, 200 Hz, and 250 Hz. For the diaphragm, the stimulations were as follows: 1 Hz (200 ms), 10 Hz (500 ms onward), 20 Hz, 40 Hz, 60 Hz, 80 Hz, 100 Hz, 125 Hz, 150 Hz, and 200 Hz. Muscle force was recorded using 610A Dynamic Muscle Control LabBook software (Aurora Scientific) and analyzed using Clampfit 10.7.0.3 software (Molecular Devices). Specific force was calculated using the wet weight of the muscle and optimized length between the proximal and distal myotendinous junctions (44, 46). A 2-way ANOVA was used to determine statistical differences between groups for frequency dependence.

### Quantification of myotonia.

Myotonia was measured using ex vivo muscle contraction. For experiments on 20-week-old mice, EDLs were isolated from WT and Ca_V_1.1^Δe29^ mice and optimized as in the previous section. EDLs were first equilibrated for 25 minutes in Ringer’s solution. Muscles were then subjected to a protocol to determine myotonia with 3 successive 200 ms, 1 Hz twitches separated by 20 seconds, a 3-minute rest period, and then 3 successive tetani stimulations involving 200 ms stimulations delivered at 150 Hz for 500 ms, separated by 3 minutes. Ringer’s solution with 100 μM 9-AC was then flowed into the bath for 25 minutes, and the myotonia protocol was repeated. This timing ensured that there was enough time for solution exchange and for the muscle to equilibrate in the new bath. To determine the impact of verapamil on myotonia in WT and Ca_V_1.1^Δe29^ muscles, the myotonia protocol (noted earlier in this section) was performed in the presence of Ringer’s solution containing either 5 μM or 20 μM verapamil, followed by Ringer’s solution containing 100 μM 9-AC and 5 μM or 20 μM verapamil. For the experiments involving 6-week-old mice, EDLs were isolated from WT, Ca_V_1.1^Δe29^, ClC-1^–/–^, and Ca_V_1.1^Δe29^ ClC-1^–/–^ mice and optimized as described in the previous section. For pretreatment recordings, muscles were equilibrated for 25 minutes in Ringer’s solution, and the myotonia protocol was performed. Ringer’s solution containing 20 μM verapamil was added to the bath using gravity flow and equilibrated for 25 minutes. The myotonia protocol was then repeated. Muscle force was recorded using 610A Dynamic Muscle Control LabBook software (Aurora Scientific), and traces were analyzed using Clampfit 10.7.0.3 software (Molecular Devices) to calculate the AUC. The AUC was then normalized to the specific force (calculated as in the previous section) and used for statistical analysis.

### Transient weakness measurements.

Transient weakness was measured in EDL muscle with ex vivo muscle contraction. EDLs from 20-week-old WT or Ca_V_1.1^Δe29^ mice were isolated and optimized as mentioned in previous sections. The experiments were contralaterally controlled, with 1 side equilibrated in normal Ringer’s solution, and the contralateral muscle was equilibrated in Ringer’s solution with 100 μM 9-AC for at least 25 minutes. Muscles were then subjected to the transient weakness protocol (45 successive 500 ms, 100 Hz tetanic pulses, separated by 4 s). To determine the effect of verapamil on transient weakness, verapamil was dissolved in Ringer’s solution and delivered to the bath by gravity flow. Using a dual-bath system, 1 muscle group received verapamil, and the contralateral muscle received verapamil plus 9-AC. To determine verapamil’s effect, we tested concentrations of both 5 μM and 20 μM and equilibrated the concentrations for 25 minutes before performing the transient weakness protocol. For experiments in mice at 6 weeks of age, EDL muscles were isolated from WT, Ca_V_1.1^Δe29^, ClC-1^–/–^, and Ca_V_1.1^Δe29^ ClC-1^–/–^ mice and equilibrated for 25 minutes in Ringer’s media containing no verapamil or 20 μM verapamil (contralaterally). The transient weakness protocol was then performed. Muscle force was recorded and analyzed as described above . Specific force was calculated, and for statistical analysis, we also quantified the percentage of the initial force generated.

### Statistics.

All averaged data are represented as the mean ± SEM. Data points collected for a single mouse are represented as open circles (1 mouse is *n* = 1), however, for ex vivo muscle contraction techniques, open circles represent 1 muscle (1 muscle is *n* = 1). The number of sampled units, *n*, for which statistics are reported is the single mouse for the in vivo experiments (1 mouse is *n* = 1). GraphPad Prism 9 (GraphPad Software) was used for statistical analyses. A 1- or 2-way ANOVA with multiple comparisons was used to test for significant differences between each of the groups tested. For survival analyses, each group was compared with each other group using the Kaplan-Meier log-rank test.

### Study approval.

All procedures in this study complied with the University of Rochester Committee on Animal Resources under the Association for Assessment and Accreditation of Laboratory Animal Care International (AAALAC) (D16-00188). All mice were housed, bred, cared for, and experimented on with the approval of the University of Rochester Committee on Animal Resources.

### Data availability.

All data are available in the main text or the supplemental materials. Values for all data points in graphs are reported in the Supplemental Supporting Data Values file.

## Author contributions

LAC, MTS, CAT, and JDL conceptualized the study. JDL designed the study methodology. LAC, MTS, KME, and JDL performed experiments. LAC and JDL acquired funding. JDL was project administrator and supervised the study. LAC, MTS, CAT, and JDL wrote the original draft of the manuscript. LAC, MTS, KME, CAT, and JDL reviewed and edited the manuscript.

## Supplementary Material

Supplemental data

Supporting data values

## Version 1 01/02/2024

Electronic publication

## References

[2] Mankodi A (2002). Expanded CUG repeats trigger aberrant splicing of ClC-1 chloride channel pre-mRNA and hyperexcitability of skeletal muscle in myotonic dystrophy. Mol Cell.

[3] Charlet BN (2002). Loss of the muscle-specific chloride channel in type 1 myotonic dystrophy due to misregulated alternative splicing. Mol Cell.

[4] Lueck JD (2007). Muscle chloride channel dysfunction in two mouse models of myotonic dystrophy. J Gen Physiol.

[5] Pang PD (2018). CRISPR -mediated expression of the fetal Scn5a Isoform in adult mice causes conduction defects and arrhythmias. J Am Heart Assoc.

[6] Freyermuth F (2016). Splicing misregulation of SCN5A contributes to cardiac-conduction delay and heart arrhythmia in myotonic dystrophy. Nat Commun.

[7] Savkur RS (2001). Aberrant regulation of insulin receptor alternative splicing is associated with insulin resistance in myotonic dystrophy. Nat Genet.

[8] Fugier C (2011). Misregulated alternative splicing of BIN1 is associated with T tubule alterations and muscle weakness in myotonic dystrophy. Nat Med.

[9] Sultana N (2016). Restricting calcium currents is required for correct fiber type specification in skeletal muscle. Development.

[10] Nakamori M (2013). Splicing biomarkers of disease severity in myotonic dystrophy. Ann Neurol.

[11] Lueck JD (2007). Chloride channelopathy in myotonic dystrophy resulting from loss of posttranscriptional regulation for CLCN1. Am J Physiol Cell Physiol.

[12] Tuluc P (2009). A CaV1.1 Ca2^+^ channel splice variant with high conductance and voltage-sensitivity alters EC coupling in developing skeletal muscle. Biophys J.

[13] Nagamitsu S (2000). A “dystrophic” variant of autosomal recessive myotonia congenita caused by novel mutations in the CLCN1 gene. Neurology.

[14] Mielke U (1985). Antimyotonic therapy with tocainide under ECG control in the myotonic dystrophy of Curschmann-Steinert. J Neurol.

[15] De Luca A (2004). New potent mexiletine and tocainide analogues evaluated in vivo and in vitro as antimyotonic agents on the myotonic ADR mouse. Neuromuscul Disord.

[16] Streib EW (1986). Successful treatment with tocainide of recessive generalized congenital myotonia. Ann Neurol.

[17] Ricker K (1978). Transient muscular weakness in severe recessive myotonia congenita. Improvement of isometric muscle force by drugs relieving myotomic stiffness. J Neurol.

[18] Rüdel R (1988). Transient weakness and altered membrane characteristic in recessive generalized myotonia (Becker). Muscle Nerve.

[19] Ricker K, Meinck HM (1972). Muscular paralysis in myotonia congenita. Eur Neurol.

[20] Myers JH (2021). The mechanism underlying transient weakness in myotonia congenita. Elife.

[21] Lund-Johansen P, Omvik P (1987). Central hemodynamic changes of calcium antagonists at rest and during exercise in essential hypertension. J Cardiovasc Pharmacol.

[22] Lee KS, Tsien RW (1983). Mechanism of calcium channel blockade by verapamil, D600, diltiazem and nitrendipine in single dialysed heart cells. Nature.

[23] Johnson BD (1996). Distinct effects of mutations in transmembrane segment IVS6 on block of L-type calcium channels by structurally similar phenylalkylamines. Mol Pharmacol.

[24] Zhao Y (2019). Molecular basis for ligand modulation of a mammalian voltage-gated Ca^2+^ Channel. Cell.

[25] Dayal A (2017). The Ca^2+^ influx through the mammalian skeletal muscle dihydropyridine receptor is irrelevant for muscle performance. Nat Commun.

[26] Wu H, Olson EN (2002). Activation of the MEF2 transcription factor in skeletal muscles from myotonic mice. J Clin Invest.

[27] Palade PT, Barchi RL (1977). On the inhibition of muscle membrane chloride conductance by aromatic carboxylic acids. J Gen Physiol.

[28] Hagiwara S, Takahashi K (1967). Surface density of calcium ions and calcium spikes in the barnacle muscle fiber membrane. J Gen Physiol.

[29] Byerly L (1985). Permeation and interaction of divalent cations in calcium channels of snail neurons. J Gen Physiol.

[30] Wang X (2023). Plateau potentials contribute to myotonia in mouse models of myotonia congenita. Exp Neurol.

[31] Cohn RD (2001). Prevention of cardiomyopathy in mouse models lacking the smooth muscle sarcoglycan-sarcospan complex. J Clin Invest.

[32] Mathieu J (1999). A 10-year study of mortality in a cohort of patients with myotonic dystrophy. Neurology.

[33] Cannon SC (2015). Channelopathies of skeletal muscle excitability. Compr Physiol.

[34] Pedersen TH (2016). Role of physiological ClC-1 Cl– ion channel regulation for the excitability and function of working skeletal muscle. J Gen Physiol.

[35] Zifarelli G, Pusch M (2010). Relaxing messages from the sarcolemma. J Gen Physiol.

[36] Tang ZZ (2012). Muscle weakness in myotonic dystrophy associated with misregulated splicing and altered gating of Ca(V)1.1 calcium channel. Hum Mol Genet.

[37] Flucher BE, Tuluc P (2017). How and why are calcium currents curtailed in the skeletal muscle voltage-gated calcium channels?. J Physiol.

[38] Rau F (2015). Abnormal splicing switch of DMD’s penultimate exon compromises muscle fibre maintenance in myotonic dystrophy. Nat Commun.

[39] Grant R (1987). Nifedipine in the treatment of myotonia in myotonic dystrophy. J Neurol Neurosurg Psychiatry.

[40] Yonekawa T (2022). *Large1* gene transfer in older *myd* mice with severe muscular dystrophy restores muscle function and greatly improves survival. Sci Adv.

[41] Beqollari D (2016). Progressive impairment of Ca_V_1.1 function in the skeletal muscle of mice expressing a mutant type 1 Cu/Zn superoxide dismutase (G93A) linked to amyotrophic lateral sclerosis. Skeletal Muscle.

[42] Bachman JF (2018). Prepubertal skeletal muscle growth requires Pax7-expressing satellite cell-derived myonuclear contribution. Development.

[43] Hakim CH (2019). An improved method for studying mouse diaphragm function. Sci Rep.

[44] Hakim CH (2011). Monitoring murine skeletal muscle function for muscle gene therapy. Methods Mol Biol.

[45] Wei-Lapierre L (2013). Orai1-dependent calcium entry promotes skeletal muscle growth and limits fatigue. Nat Commun.

[46] Hakim CH (2013). Evaluation of muscle function of the extensor digitorum longus muscle ex vivo and tibialis anterior muscle in situ in mice. J Vis Exp.

