# Supplementary material for: Verapamil mitigates chloride and calcium bi-channelopathy in a myotonic dystrophy mouse model
Source: J Clin Invest. 2024 Jan 2;134(1):e173576. doi: 10.1172/JCI173576 (PMC10760957; doi:10.1172/JCI173576)
Supplement: Supplemental data [file jci-134-173576-s143.pdf]

Supplemental information for "Verapamil mitigates chloride and calcium bi-channelopathy in a myotonic dystrophy mouse model"

Lily A. Cisco[1], Matthew T. Sipple[1], Katherine M. Edwards[1], Charles A. Thornton[2,3] and John D. Lueck[1,2,3,*]

Corresponding author: john_lueck@urmc.rochester.edu

The PDF file includes:

Supplemental Figs. 1 to 13
Supplemental Tables 1-5

Supplemental Figure 1

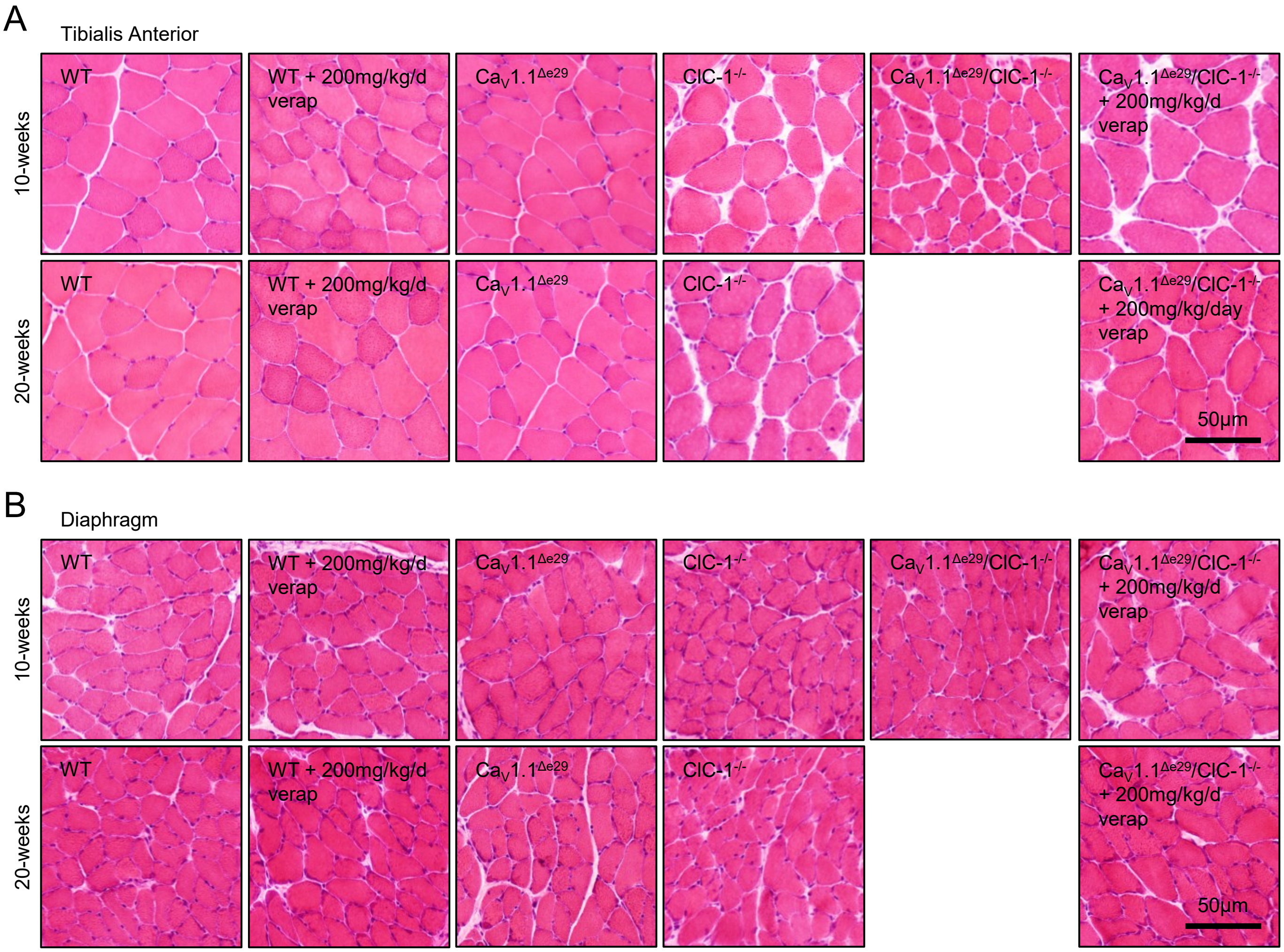

Supplemental Figure 1. Ca$_V$1.1$^{\Delta e29}$/ClC-1$^{-/-}$ genotype does not impart overt dystrophic histological features in limb or diaphragm muscle. (**A**) Hematoxylin and Eosin staining of 10µm transverse sections of snap frozen tibialis anterior from 10-weeks (top) and 20-weeks (bottom) tissue samples. (**B**) Hematoxylin and Eosin staining of 10µm transverse sections of snap frozen diaphragm from 10-weeks (top) and 20-weeks (bottom) tissue samples.

Supplemental Figure 2

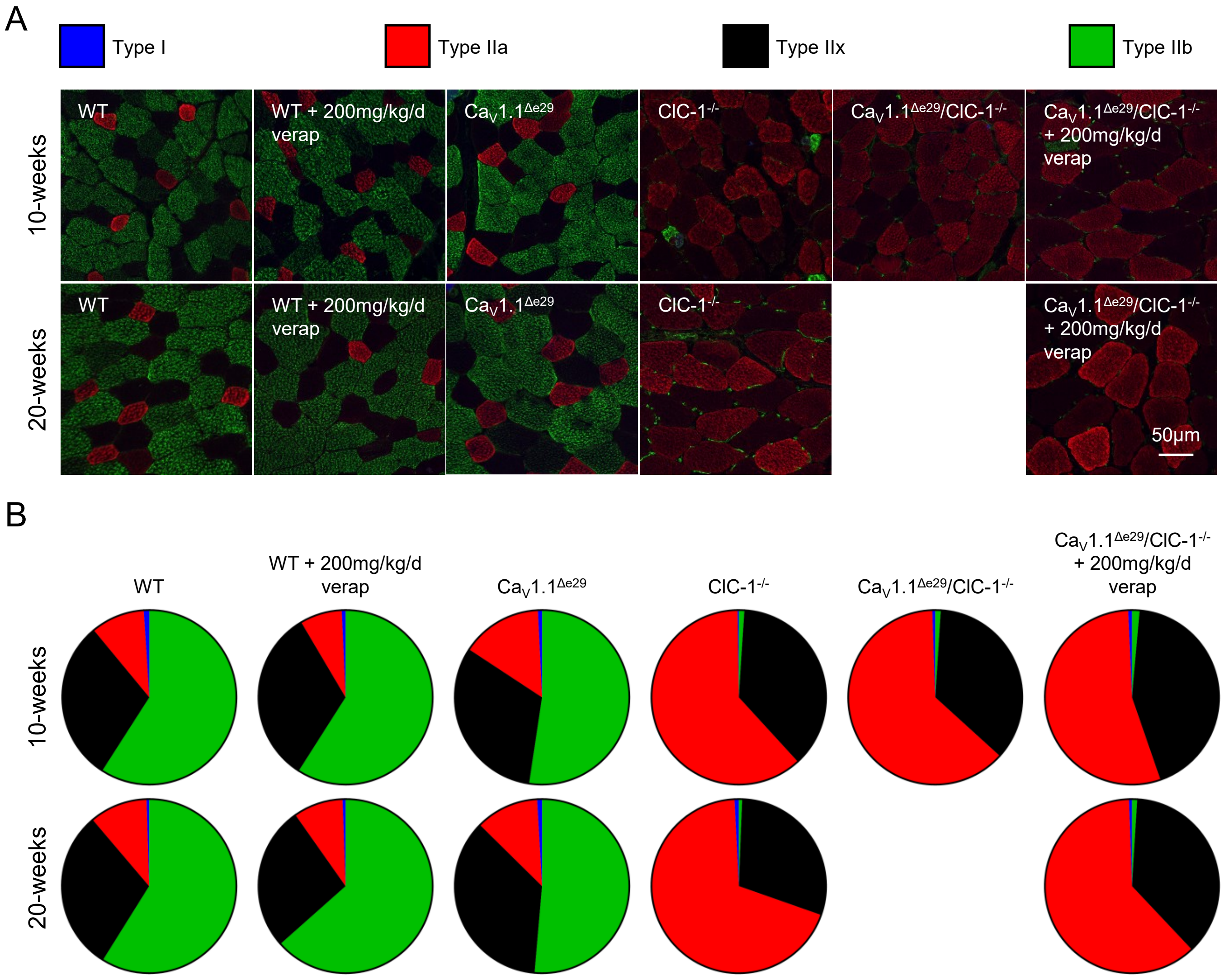

Supplemental Figure 2. $Ca_V1.1^{\Delta e29}$/ClC-$1^{-/-}$ limb muscle fiber type distribution is not altered from ClC-$1^{-/-}$. (**A**) Representative images of fiber-type immunostaining for 10-weeks (top) and 20-weeks (bottom) tibialis anterior muscle isolated from indicated genotype and treatment groups. MYHC type IIB fibers, green; IIX, black; IIA, red; I, blue. Scale bars: 50μm. (n=5/group). (**B**) Average quantification of fiber-type percentages of tibialis anterior muscle isolated from indicated genotype and treatment groups. Type IIB fibers (green), IIX, (black) IIA, (red) I, (blue). Scale bars: 50μm. (n=5/group)

Supplemental Figure 3

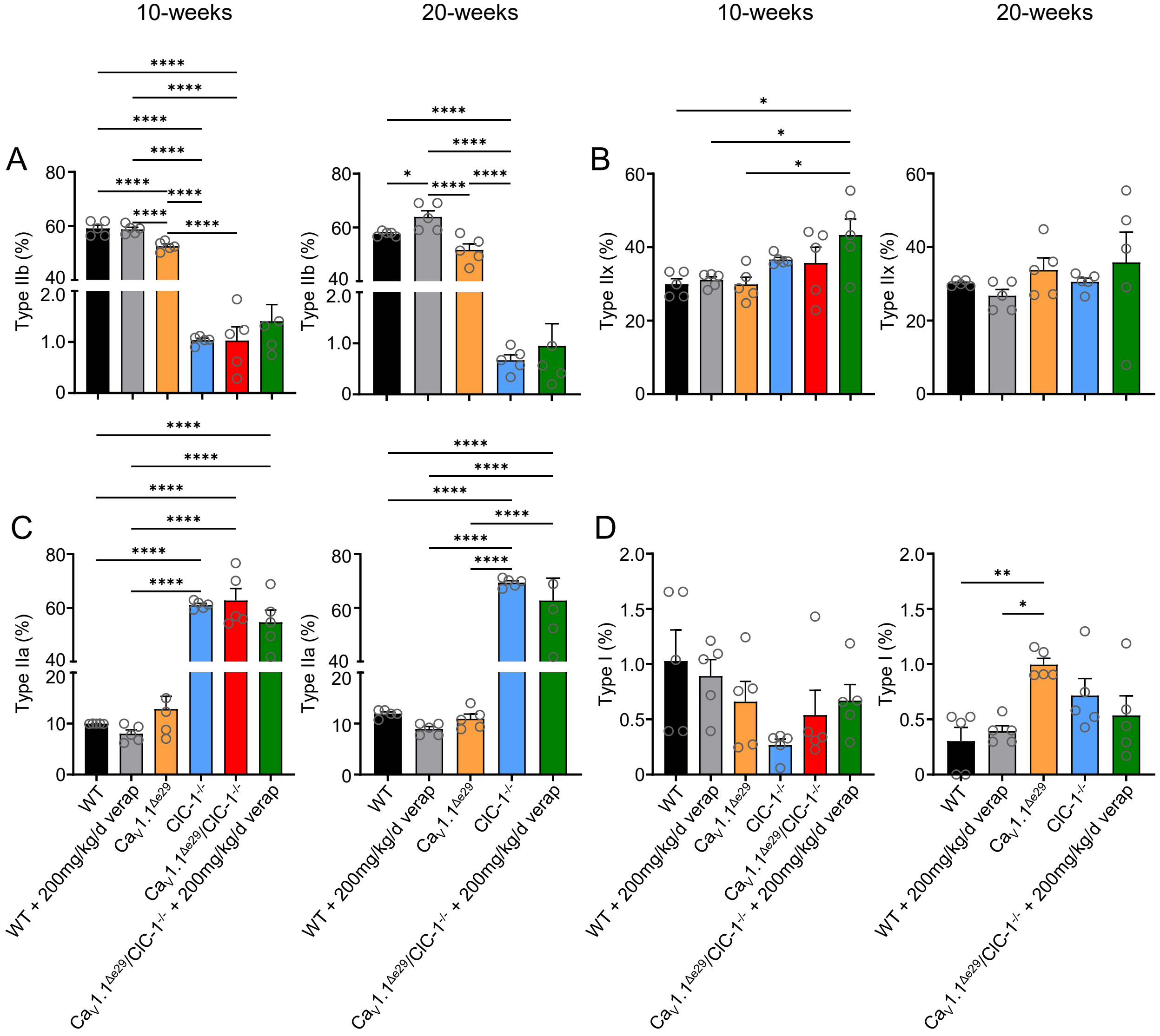

Supplemental Figure 3. Quantification and statistical analysis of fiber type distribution of tibialis anterior muscle. (**A**-**D**) Quantification of (**A**) type IIb (**B**) type IIx (**C**) type IIa (**D**) type I fibers for 10-weeks (left) and 20-weeks (right) tibialis anterior (n=5/group). One-way ANOVA with Tukey's post-hoc analysis, * = $P < 0.05$, ** = $P < 0.01$, *** = $P < 0.001$, and **** = $P < 0.0001$.

Supplemental Figure 4

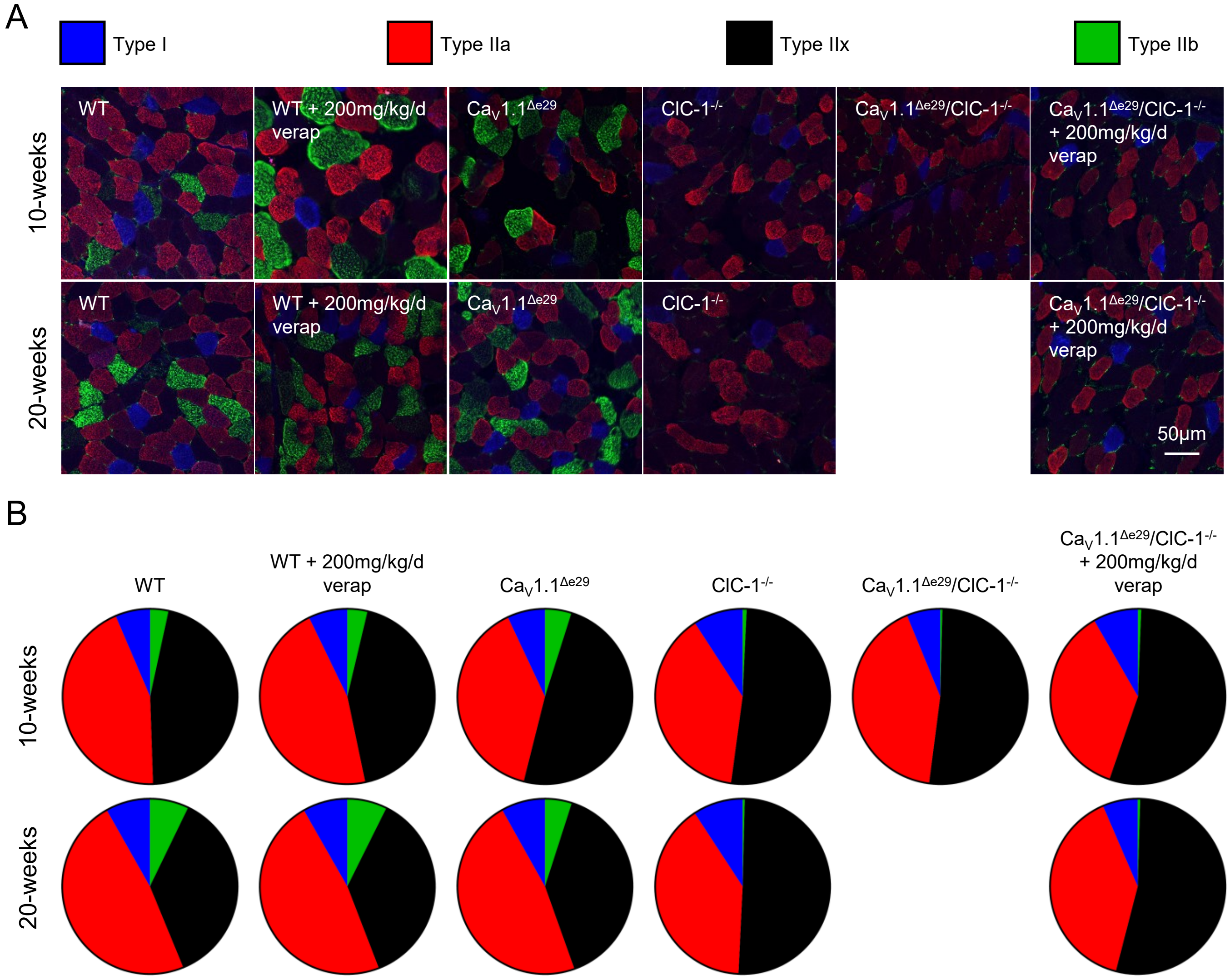

Supplemental Figure 4. $Ca_V1.1^{\Delta e29}$/ClC-$1^{-/-}$ diaphragm muscle fiber type distribution is not altered from ClC-$1^{-/-}$. (**A**) Representative images of fiber-type immunostaining for 10-weeks (top) and 20-weeks (bottom) diaphragm muscle isolated from indicated genotype and treatment groups. MYHC type IIB fibers, green; IIX, black; IIA, red; I, blue. Scale bars: 50 µm. (n=5/group). (**B**) Average quantification of fiber-type percentages of diaphragm muscle isolated from indicated genotype and treatment groups. Type IIB fibers (green), IIX, (black) IIA, (red) I, (blue). Scale bars: 50 µm. (n=5/group)

Supplemental Figure 5

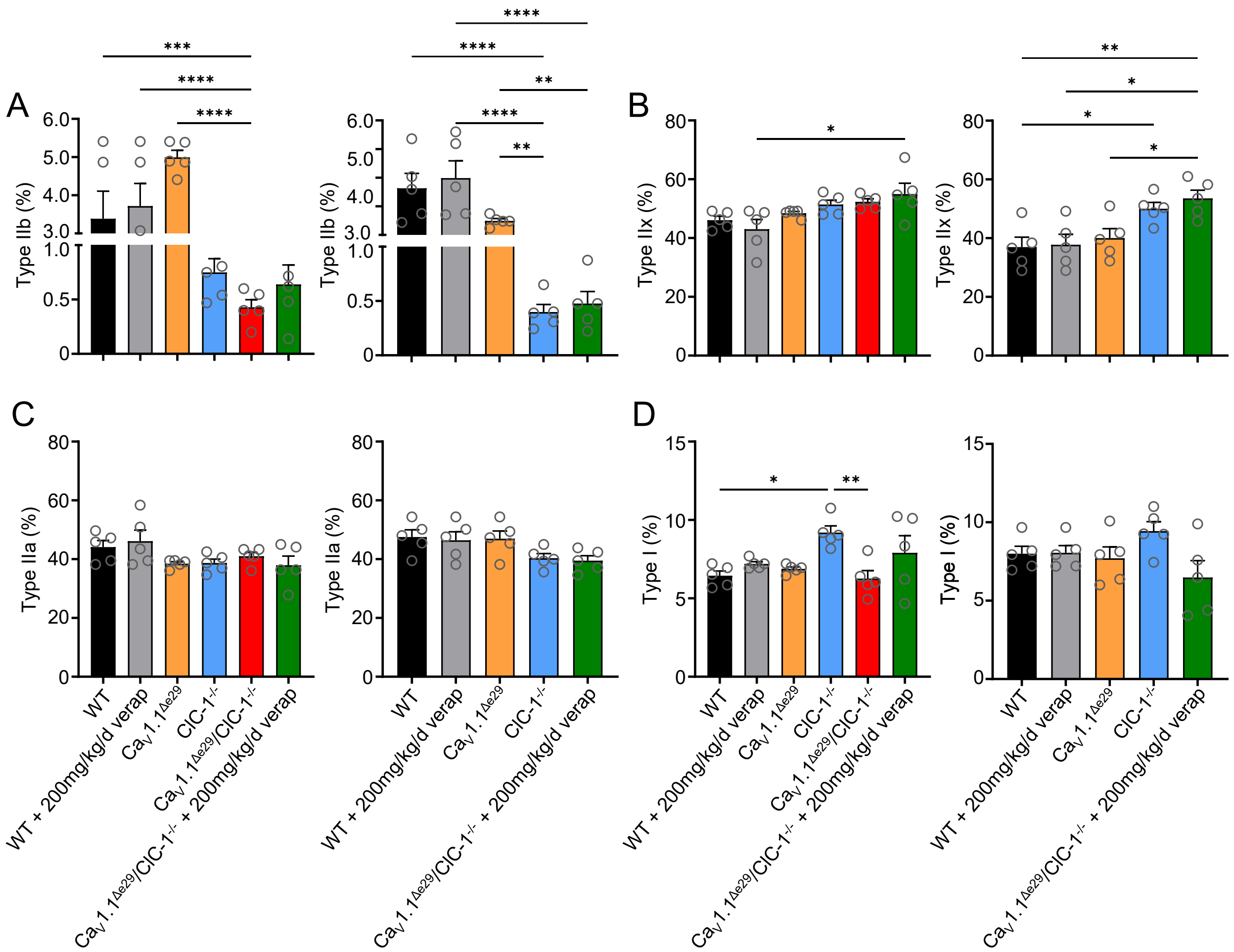

Supplemental Figure 5. Quantification and statistical analysis of fiber type distribution of diaphragm muscle. (**A**-**D**) Quantification of (**A**) type IIb (**B**) type IIx (**C**) type IIa (**D**) type I fibers for 10-weeks (left) and 20-weeks (right) diaphragm (n=5/group). One-way ANOVA with Tukey's post-hoc analysis, * = P < 0.05, ** = P < 0.01, *** = P < 0.001, and **** = P < 0.0001.

Supplemental Figure 6

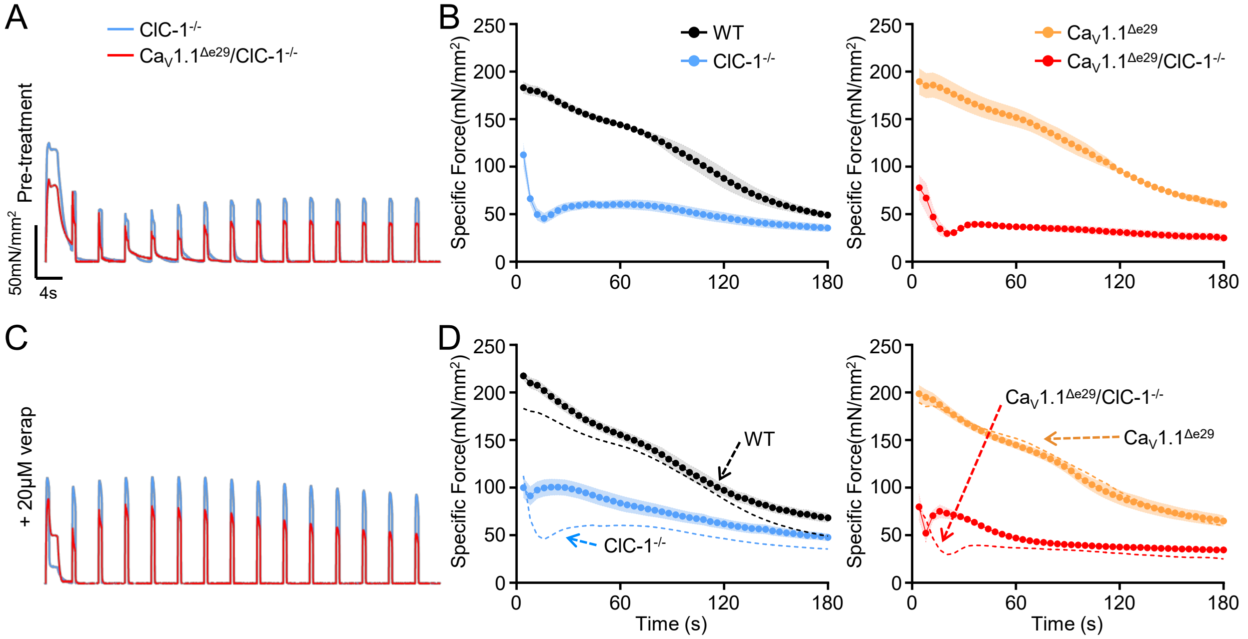

Supplemental Figure 6. Ca$_V$1.1$^{\Delta e29}$/ClC-1$^{-/-}$ muscle exhibits severe transient weakness that is significantly improved by the addition of verapamil (not normalized to the first peak force). (**A** and **C**) Representative specific force traces of the first 15 tetani (100Hz, 500ms) separated by four seconds, recorded *ex vivo* from EDLs isolated from 6-week ClC-1$^{-/-}$ (blue) and Ca$_V$1.1$^{\Delta e29}$/ClC-1$^{-/-}$ (red) mice in the (**A**) absence and (**C**) presence of 20μM verapamil added to the bath. (**B** and **D**) Plot of the average peak tetanic EDL, elicited by 44 subsequent 100Hz, 500ms tetanic stimulations separated by four seconds from 6-week WT (black, n=4), ClC-1$^{-/-}$ (blue n=4), Ca$_V$1.1$^{\Delta e29}$ (orange, n=4) and Ca$_V$1.1$^{\Delta e29}$/ClC-1$^{-/-}$ red, n=4) mice in the (**B**) absence and (**D**) presence of 20μM verapamil added to the bath for ClC-1$^{-/-}$ (blue n=4) and Ca$_V$1.1$^{\Delta e29}$/ClC-1$^{-/-}$ red, n=4) EDLs. Dashed lines in (**D**) represent average data presented in (**B**) as a reference for pre-treatment. Symbols, closed circles, mean ± SEM. Statistical analysis of results in Supplemental Figure 6 are found in Supporting Data. (**B** and **D**) Two-way ANOVA with Tukey's post-hoc analysis.

Supplemental Figure 7

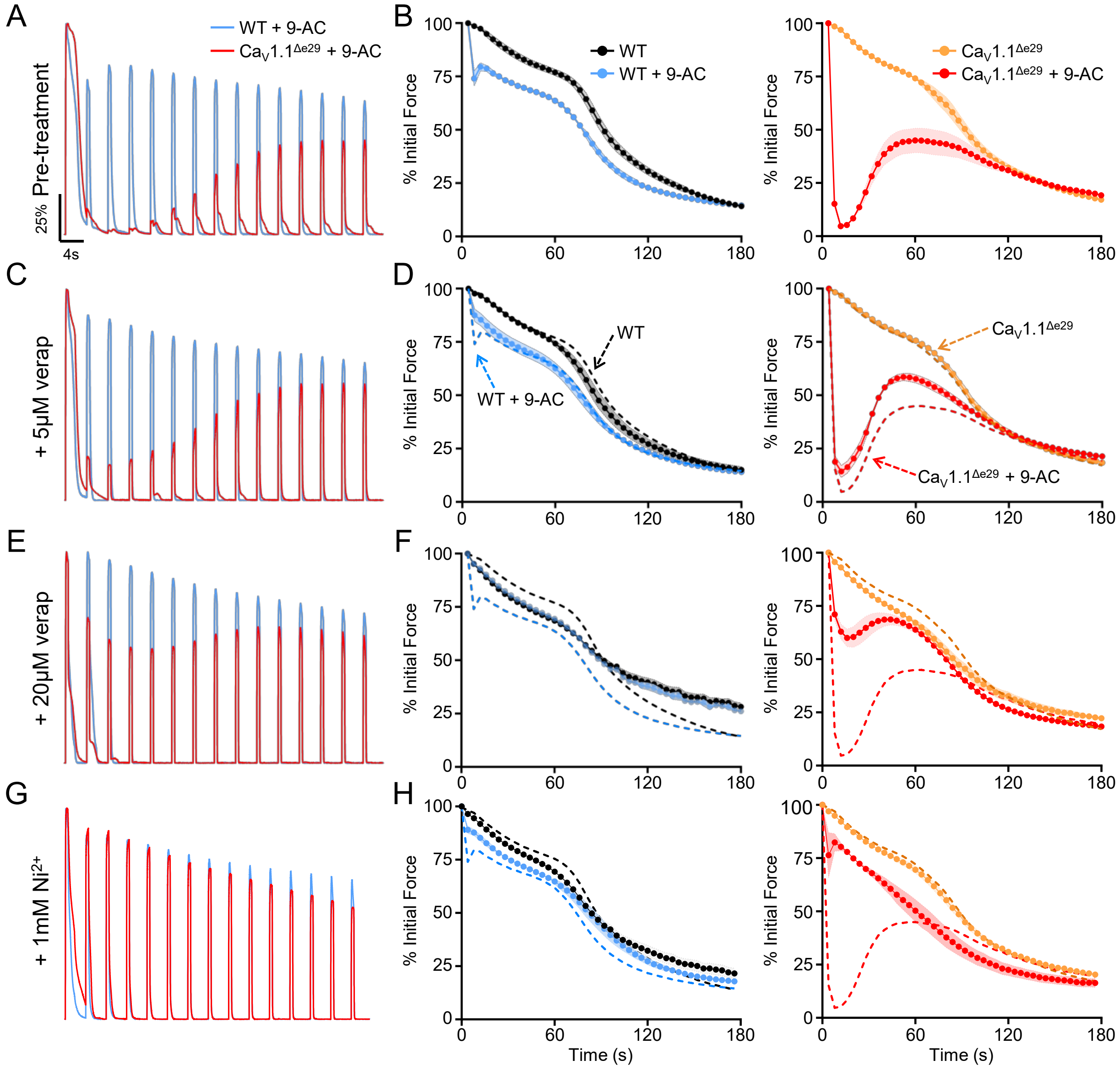

Supplemental Figure 7. Ca$_V$1.1$^{\Delta e29}$ exacerbates transient weakness in myotonic muscle and is alleviated by verapamil. (**A**) Normalized representative force traces of the first 15 tetani (100Hz, 500ms) separated by four seconds, recorded *ex vivo* from EDLs from 20-wk WT (blue) and Ca$_V$1.1$^{\Delta e29}$ (red) mice in the presence of 100µM 9-AC (pre-treatment). (**B**) Average peak tetanic EDL forces normalized to the initial stimulus, elicited by 44 subsequent 100Hz, 500ms tetanic stimulations separated by four seconds from WT (black), WT + 9-AC (blue), Ca$_V$1.1$^{\Delta e29}$ (orange) and Ca$_V$1.1$^{\Delta e29}$ + 9-AC (red) mice. (**C**, **E,** and **G**) Normalized representative force traces of the first 15 tetani recorded *ex vivo* from EDLs from 20-wk WT (blue) and Ca$_V$1.1$^{\Delta e29}$ (red) mice in the presence of 100µM 9-AC and (**C**) 5µm verapamil**,** (**E**) 20µM verapamil, (**G**) or 1mM Ni^{2+}. (**D**). Average peak tetanic forces normalized to the initial stimulus from WT + 5µM verapamil (black), WT + 9-AC + 5µM verapamil (blue), Ca$_V$1.1$^{\Delta e29}$ + 5µM verapamil (orange) and Ca$_V$1.1$^{\Delta e29}$ + 9-AC + 5µM verapamil (red) EDLs. (**F**) Average peak tetanic forces normalized to the initial stimulus from WT + 20µM verapamil (black), WT + 9-AC + 20µM verapamil (blue), Ca$_V$1.1$^{\Delta e29}$ + 20µM verapamil (orange) and Ca$_V$1.1$^{\Delta e29}$ + 9-AC + 20µM verapamil (red) EDLs. (**H**) Average peak tetanic EDL forces normalized to the initial stimulus from WT + 1mM Ni^{2+} (black), WT + 9-AC + 1mM Ni^{2+} (blue), Ca$_V$1.1$^{\Delta e29}$ + 1mM Ni^{2+} (orange) and Ca$_V$1.1$^{\Delta e29}$ + 9-AC + 1mM Ni^{2+} (red) EDLs. Dashed lines in (**D**, **F**, and **H**) represent average data from (**B**) for pre-treatment reference. Symbols, closed circles, mean ± SEM. n=5 for all experimental groups. Note: Contralateral EDLs were used. Statistical analysis of results in Supplemental Figure 7 are found in Supporting Data. (**B**, **D**, **F** and **H**) Two-way ANOVA with Tukey's post-hoc analysis.

Supplemental Figure 8

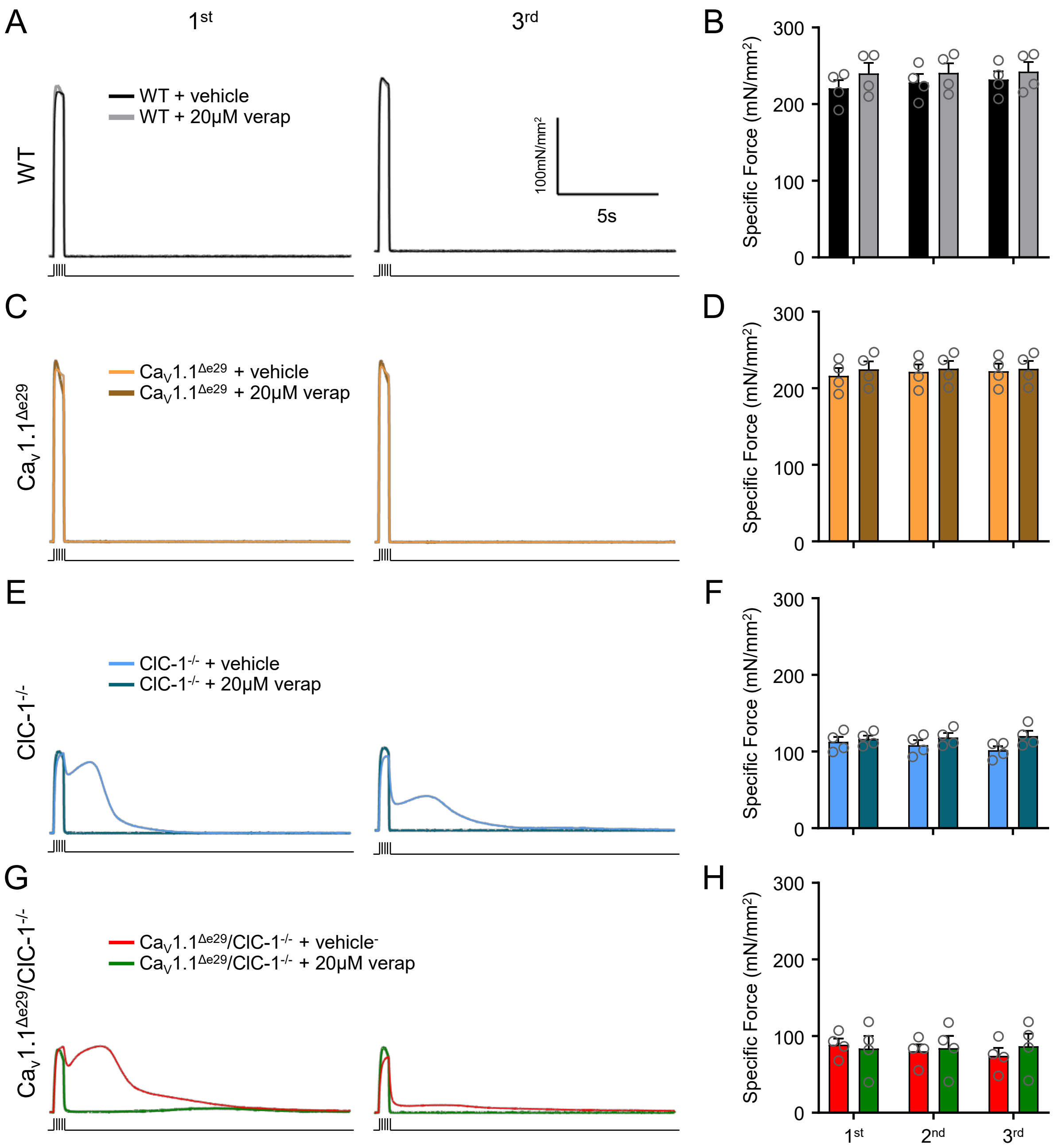

Supplemental Figure 8. Verapamil treatment does not reduce peak contraction force of WT, $Ca_V1.1^{\Delta e29}$, ClC-1$^{-/-}$ and $Ca_V1.1^{\Delta e29}$/ClC-1$^{-/-}$ mouse muscle. (**A, C, E,** and **G**) Representative traces of the first (left) and third (right) tetani (150Hz, 500ms) in (**A**) WT, (**C**) $Ca_V1.1^{\Delta e29}$, (**E**) ClC-1$^{-/-}$ and (**G**) $Ca_V1.1^{\Delta e29}$/ClC-1$^{-/-}$ EDLs in the absence and presence of 20μM verapamil. Treatment depicted by colors defined in legends. (**B, D, F,** and **H**) Average specific force for (**B**) WT (**D**) $Ca_V1.1^{\Delta e29}$ (**F**) ClC-1$^{-/-}$ and (**H**) $Ca_V1.1^{\Delta e29}$/ClC-1$^{-/-}$ EDLs across 3 tetanic stimulations in the absence and presence of 20μM verapamil. Treatment depicted by colors defined in legends. Symbols, open circles, individual mice; bars, mean and SEM. Statistical analysis of results in Supplemental Figure 8 are found in Supporting Data. (**B**, **D, F** and **H**) Two-way ANOVA with Tukey's post-hoc analysis.

Supplemental Figure 9

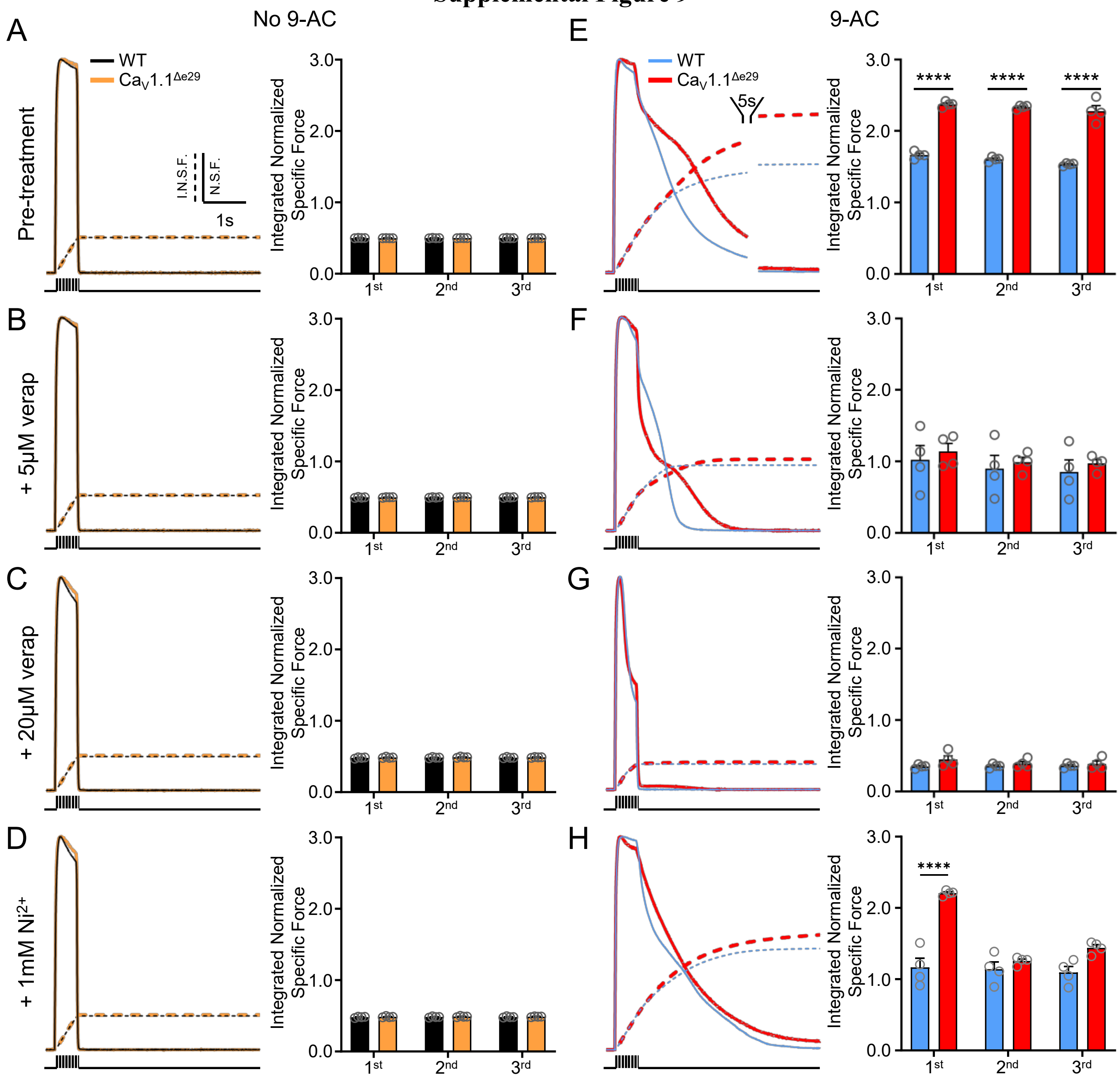

Supplemental Figure 9. Ca$_V$1.1$^{\Delta e29}$ significantly exacerbates myotonia. (**A, B, C,** and **D** left) Normalized representative force traces of the third of three tetani (100Hz, 500ms) separated by 3 minutes, recorded *ex vivo* from EDLs isolated from 20-wk WT (black) and Ca$_V$1.1$^{\Delta e29}$ (orange) mice in the (**A,** left**)** absence (pre-treatment) and presence of (**B,** left) 5µM verapamil, (**C,** left) 20µM verapamil, (**D,** left) or 1mM Ni^{2+} added to the bath (pre-treatment). Dashed lines represent accumulated force production. (**A, B**, **C,** and **D** right) Plot of average integration normalized to specific force depicted in respective left panels. (**D, E, F,** and **G** left**)** Normalized representative force traces of the third of three tetani (100Hz, 500ms) separated by 3 minutes, recorded *ex vivo* from EDLs incubated with 100µM 9-AC, isolated from 20-wk WT (blue) and Ca$_V$1.1$^{\Delta e29}$ (red) mice in the (**E,** left) absence (pre-treatment) and presence of (**F,** left) 5µM verapamil, (**G,** left) 20µM verapamil, (**H, left**) or 1mM Ni^{2+} added to the bath (pre-treatment). Dashed lines represent accumulated force production. (**E, F, G,** and **H** right) Plot of average integration normalized to specific force depicted in respective left panels. Symbols, open circles, individual mice; bars, mean and SEM. Note: Contralateral EDLs were used when possible. Statistical analysis of results in Supplemental Figure 9 are found in Supporting Data. Two-way ANOVA with Tukey's post-hoc analysis.

Supplemental Figure 10

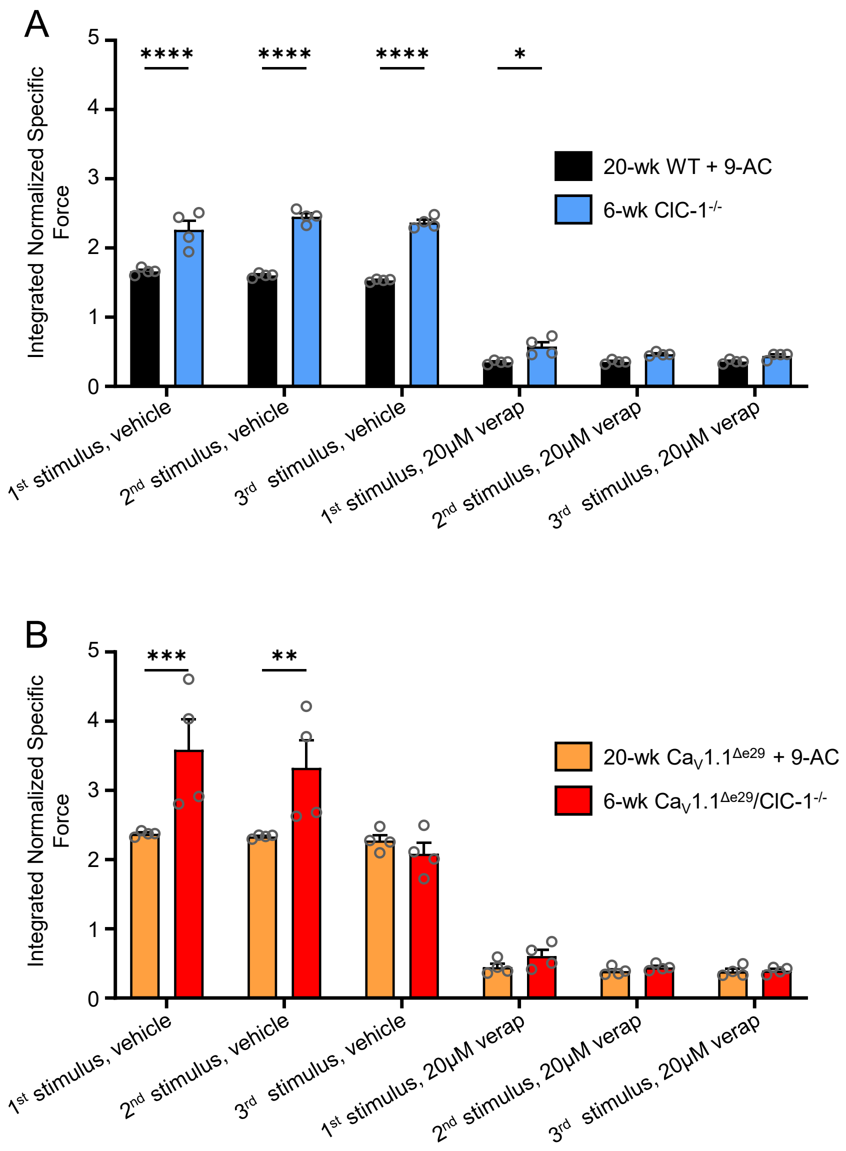

Supplemental Figure 10. Comparison of pharmacologic and genetic myotonia. (**A**) Plot of average integration normalized to specific force of WT EDL + 9-AC (black) and ClC-1$^{-/-}$ (blue). (**B**) Plot of average integration normalized to specific force of $Ca_V1.1^{\Delta e29}$ EDL + 9-AC (orange) and $Ca_V1.1^{\Delta e29}$/ClC-1$^{-/-}$ (red). Two-way ANOVA with Tukey's post-hoc analysis, * = P < 0.05, ** = P < 0.01, *** = P < 0.001, and **** = P < 0.0001

Supplemental Figure 11

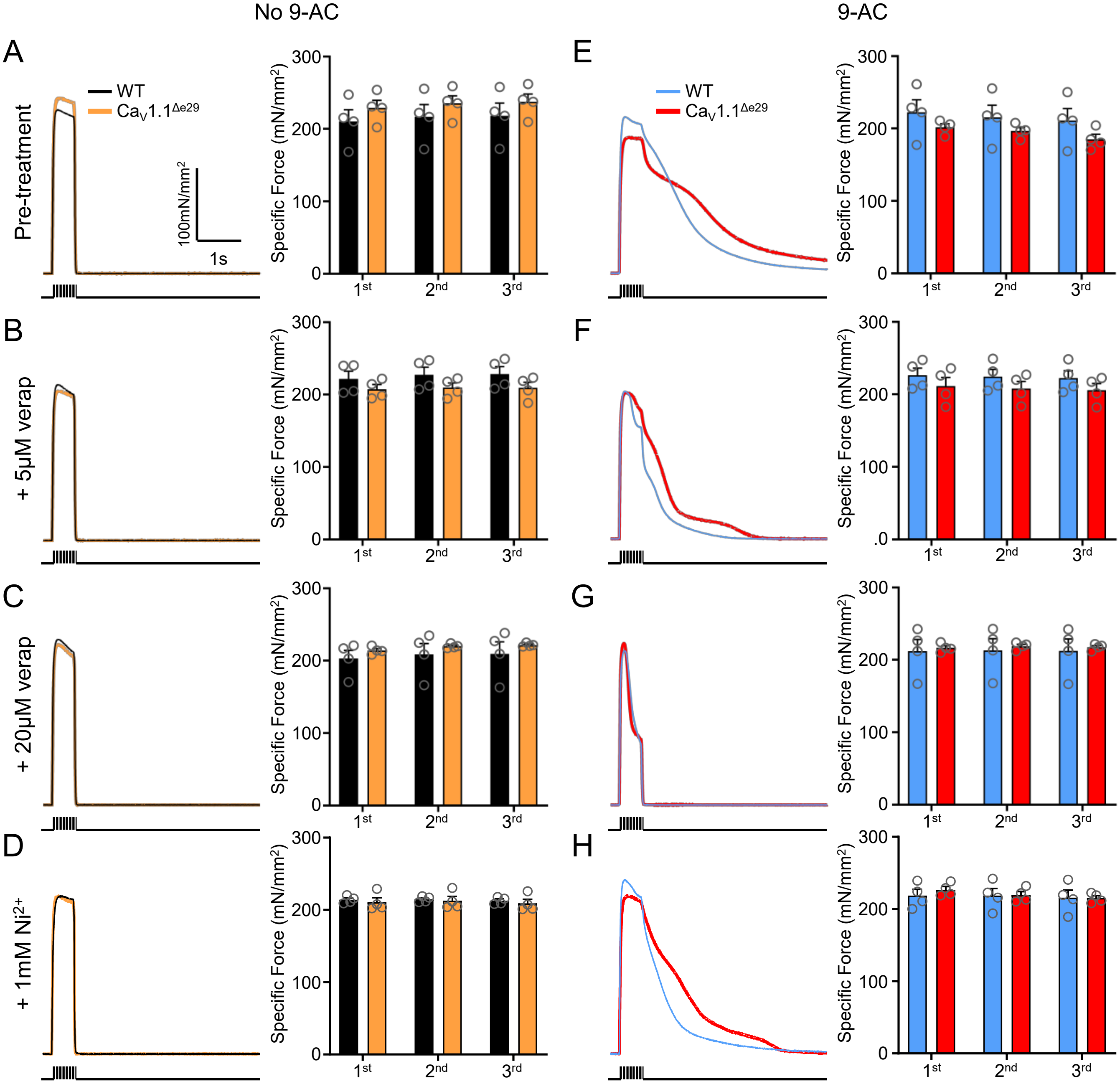

Supplemental Figure 11. Verapamil treatment does not reduce peak contraction force of non-myotonic and myotonic WT and $Ca_V1.1^{\Delta e29}$ mouse muscle. Representative specific force traces of the third of three tetani (100Hz, 500ms) separated by 3 minutes, recorded *ex vivo* from EDLs isolated from 20-wk WT (black) and $Ca_V1.1^{\Delta e29}$ (orange) mice in the (**A,** left**)** absence (pre-treatment) and presence of (**B,** left**)** 5µM verapamil, (**C,** left**)** 20µM verapamil, (**D**, left) or 1mM Ni^{2+} added to the bath (pre-treatment). Dashed lines represent accumulated force production. (**A, B, C,** and **D** right) Plot of average integration of specific force depicted in respective left panels. (**E, F, G** and **H,** left**)** Representative specific force traces of the third of three tetani (100Hz, 500ms) separated by 3 minutes, recorded *ex vivo* from EDLs incubated with 100µM 9-AC, isolated from 20-wk WT (blue) and $Ca_V1.1^{\Delta e29}$ (red) mice in the (**E,** left**)** absence (pre-treatment) and presence of (**F,** left**)** 5µM verapamil, (**G,** left**)** 20µM verapamil, (**H**, left) or 1mM Ni^{2+} added to the bath (pre-treatment). Symbols, open circles, individual mice; bars, mean and SEM. Note: Contralateral EDLs were used when possible. Statistical analysis of results in Supplemental Figure 11 are found in Supporting Data. Two-way ANOVA with Tukey's post-hoc analysis.

Supplemental Figure 12

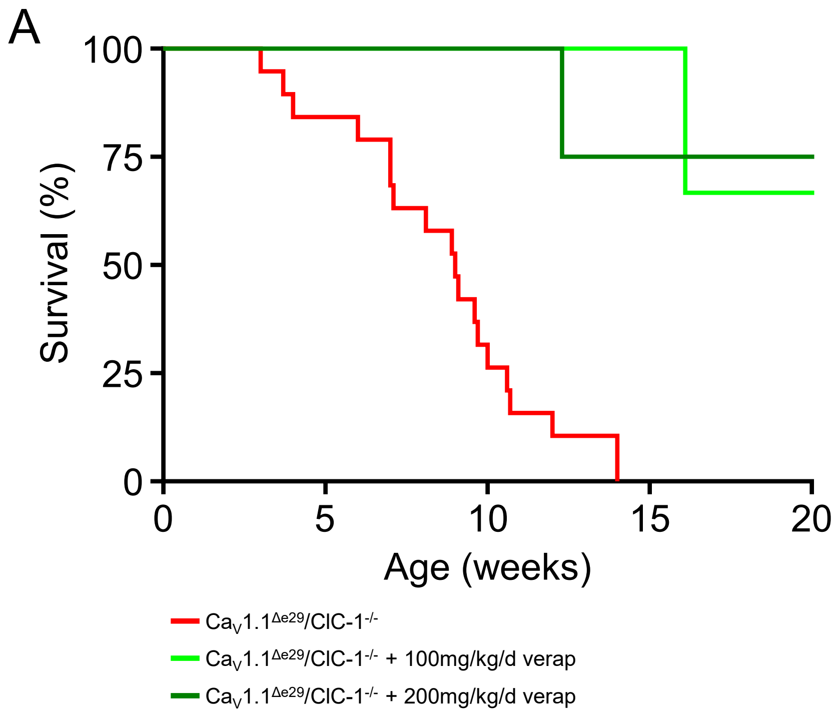

Supplemental Figure 12. Trial of two doses of verapamil in $Ca_V1.1^{\Delta e29}$/ClC-1$^{-/-}$ mice results in significant rescue of survival. (**A**) Kaplan-Meier survival analysis of $Ca_V1.1^{\Delta e29}$/ClC-1$^{-/-}$ (n=19; female=9, male=10), $Ca_V1.1^{\Delta e29}$/ClC-1$^{-/-}$ + 100mg/kg/day verapamil (n=3; female=1, male=2), and $Ca_V1.1^{\Delta e29}$/ClC-1$^{-/-}$ + 200mg/kg/day verapamil (n=4; female=2, male=2). Verapamil is dosed in mouse nutrition/hydration food cups. Statistical analysis of results in Supplemental Figure 13 are found in Supporting Data. Log-rank analysis.

Supplemental Figure 13

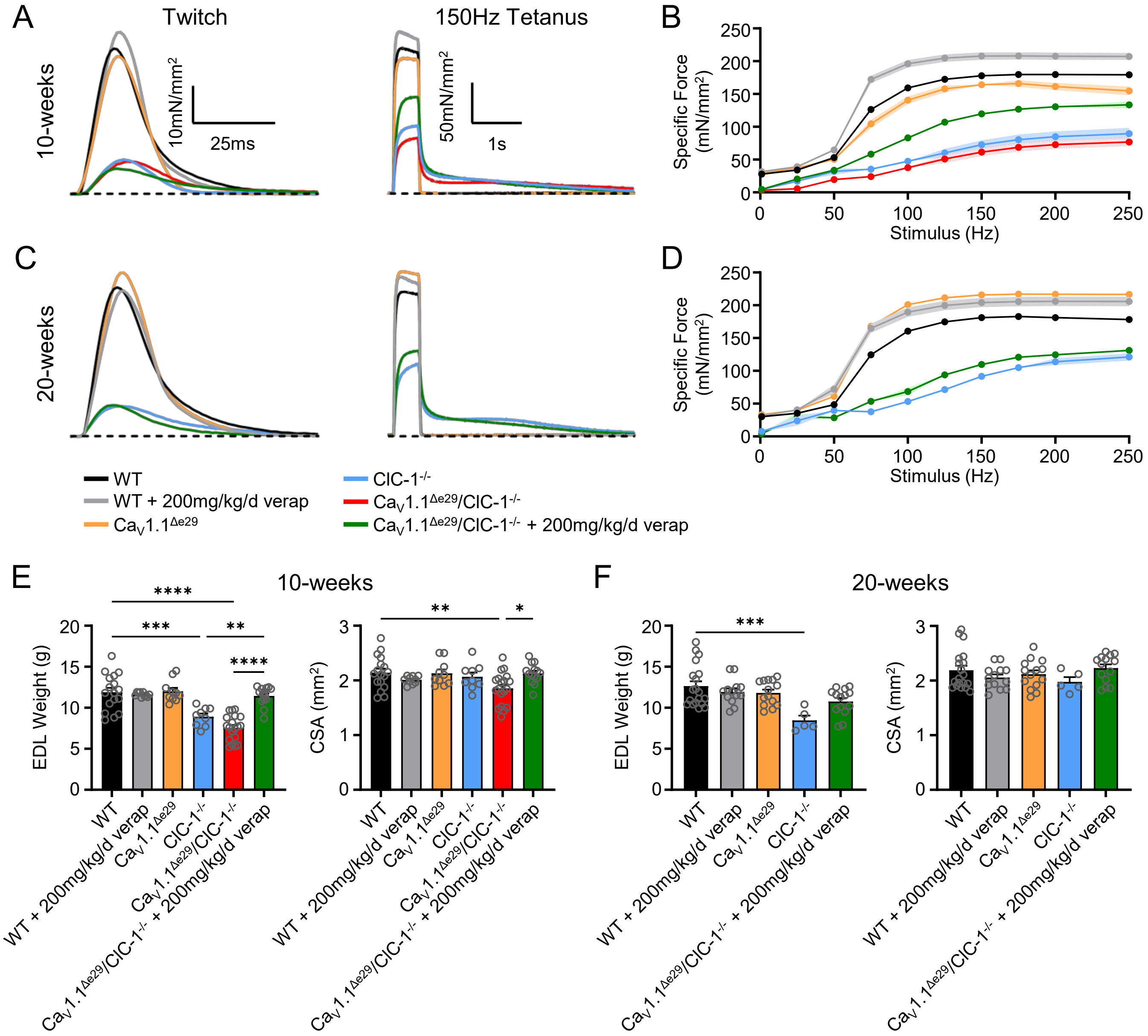

Supplemental Figure 13. Verapamil treatment improves survival, body weight, and muscle function in Ca$_V$1.1$^{\Delta e29}$/ClC-1$^{-/-}$ mice. (**A** and **C**) Representative specific force traces elicited by twitch (left) and 150Hz (500ms) tetanic (right) stimulation of EDL muscle isolated from (**A**) 10-week and (**C**) 20-week mice. (**B, D**) Plot of average stimulation frequency dependence of specific force generation from isolated EDL muscles at (**D**) 10-weeks and (**F**) 20-weeks of age in the indicated genotype and treatment groups. (**E**) 10-week and (**F**) 20-week EDL weights (left) and cross-sectional area (CSA; right). (**B** and **E**) "n" represents individual EDLs, WT (n=17; female=8, male=9), WT + 200mg/kg/day verapamil (n=10; female=5, male=5) Ca$_V$1.1$^{\Delta e29}$ (n=10; female=5, male=5), ClC-1$^{-/-}$ (n=9; female=4, male=5), Ca$_V$1.1$^{\Delta e29}$/ClC-1$^{-/-}$ (n=19; female=8, male=11), and Ca$_V$1.1$^{\Delta e29}$/ClC-1$^{-/-}$ + verapamil (n=14, female=7, male=7). (**D** and **F**) "n" represents individual EDLs, WT (n=19; female=10, male=9), WT + 200mg/kg/day verapamil (n=13; female=6, male=7), Ca$_V$1.1$^{\Delta e29}$ (n=14; female=7, male=7), ClC-1$^{-/-}$ (n=5; female=3, male=2), Ca$_V$1.1$^{\Delta e29}$/ClC-1$^{-/-}$ + verapamil (n=14; female=7, male=7). Symbols, open circles, individual mice (**B**) or individual EDLs (**G** and **H**); closed circles, means ± SEM. Statistical analysis of results in Figure 13 are found in Supporting Data. (**B** and **D**) Two-way and (**E** and **F**) one-way ANOVA with Tukey's post-hoc analysis.

Supplemental Table 1: Modified Boltzmann Fitting Parameters for Figure 2

Genotype:	G_{max} (pS/pF)	V_{rev} (mV)	$V_{1/2\ max}$ (mV)	K_{act} (mV)
WT (C57Bl/6)	0.224 ±0.011	74.897 ±1.485	4.568 ±0.758	4.350 ±0.530
$Ca_V1.1^{\Delta E29/+}$	0.332 ±0.017****	71.228 ±2.052	-18.128 ±1.100****	2.221 ±1.936
$Ca_V1.1^{\Delta E29/\Delta E29}$	0.308 ±0.020**	70.679 ±2.626	-19.019 ±1.077****	2.220 ±1.145
Statistics (P-value)	<0.0001	0.2863	<0.0001	0.3793

All data presented mean ± SE; Standard One-Way ANOVA analysis P-value reported; * indicate significant differences compared to wildtype (2-sample t-test with Turkey's correction for multiple comparisons). No significant differences between homozygote ($Ca_V1.1^{\Delta e29/\Delta e29}$) and heterozygote ($Ca_V1.1^{\Delta e29/+}$) recordings were observed.

Supplemental Table 2: sgRNA sequences for generation of force splice variant mice

Transcript	Guide	Sequence
$Ca_V1.1$	Forward	5'- GACCTCATGTGGCCGCAGTC AGG -3'
$Ca_V1.1$	Reverse	5'- GAGCCCCGAGAAATGGGTTG AGG -3'
RyR1	Forward	5' CTGGGGCTCTCTGTCGGGCTGGG 3'
RyR1	Reverse	3' ACAGGGGGTTTGAAAGGGTGGGG 5'
SERCA1	Forward	5'- CCACTCCAGCTATGACTGGT GGG -3'
SERCA1	Reverse	5'- GCGCGCGCAAGTGACCGCAG GGG -3

Supplemental Table 3: Validation of exon 29 removal from $Ca_V1.1$ by RT-PCR

Transcript	Direction	Exon	Sequence
$Ca_V1.1$	Forward	27	5’ CCAGTCGGAACAGATGAACCAC 3’
$Ca_V1.1$	Reverse	31	5’ CCGATGACCGCGTAGATGAAGA 3’
RyR1	Forward	66	5’ CCGAATCATTGTGAACAACCTGG 3’
RyR1	Reverse	72	5’ GAAGGAATTCACGGACCTCCTC 3’
SERCA1	Forward	18	5’ TGGGTGCAGCCACTGTAGGAG 3’
SERCA1	Reverse	23	5’ AAGGGTCAGTGCCTCAGCTTTG 3’

Supplemental Table 4: Immunohistochemistry

Primary Antibody	Concentration	Catalogue No.	Supplier
Myosin heavy chain Type I (Isotype: MIgG2b)	1:40	BA-D5	Developmental Studies Hybridoma Bank
Myosin heavy chain Type IIA (Isotype: MIgG1)	1:40	SC-71	Developmental Studies Hybridoma Bank
Myosin heavy chain Type IIB (Isotype: MIgM)	1:40	BF-F3	Developmental Studies Hybridoma Bank
**Primary Antibody**	**Concentration**	**Catalogue No.**	**Supplier**
AffiniPure Fab Fragment Goat Anti-Mouse IgG (H+L)	3:100	115-007-003	Jackson ImmunoResearch
**Secondary Antibody**	**Concentration**	**Catalogue No.**	**Supplier**
Goat anti-Mouse IgM (Heavy chain) Cross-Adsorbed Secondary Antibody, Alexa Fluor™ 488	1:1500	A-21042	Invitrogen
Goat anti-Mouse IgG1 Cross-Adsorbed Secondary Antibody, Alexa Fluor™ 568	1:1500	A-21124	Invitrogen
DyLight™ 405 AffiniPure Fab Fragment Goat Anti-Mouse IgG2b, Fcγ fragment specific	1:1000	115-477-187	Jackson ImmunoResearch

Supplemental Table 5: Calcium Channel Blocker Used

Drug	Catalogue No.	Supplier
(±)-verapamil hydrochloride	V4629	Sigma-Aldrich
